# Protein binding regulates complex configuration: comparative analysis of three dynamically racemic europium(iii) complexes

**DOI:** 10.1039/d6ra02760a

**Published:** 2026-05-18

**Authors:** Huishan Li, Dominic J. Black, Robert Pal, David Parker

**Affiliations:** a Department of Chemistry, Hong Kong Baptist University Kowloon Tong Hong Kong SAR China davidparker@hkbu.edu.hk; b Department of Chemistry, Durham University South Road Durham DH1 3LE UK

## Abstract

The binding behaviour of three dynamically racemic Eu(iii) complexes of heptadentate ligands based on triazacyclononane has been examined by luminescence and CPL spectroscopy. The low water solubility of the europium complexes is shown to be related to their tendency to oligomerise in aqueous media *via* intermolecular carboxylate ligation. Aqueous solubility is enhanced in the presence of hydrogencarbonate, with which a more stable and water soluble 1 : 1 ternary adduct reversibly forms. In one case, the presence of human serum albumin leads to selective complexation of the Λ isomer, while bovine serum albumin favours binding of the Δ enantiomer, as deduced by signature CPL spectra.

## Abstract 2

Induced Eu CPL signatures of opposite sign show that the same Eu(iii) complex binds predominantly as the Λ enantiomer to human serum albumin, and as the Δ isomer to bovine serum albumin.

## Graphical abstract

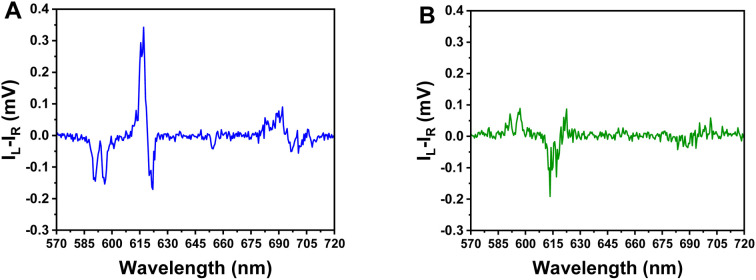

## Introduction

The design of a chiral lanthanide probe normally requires the use of complexes with well-defined solution-phase speciation. This design criterion is essential for establishing reliable correlations between molecular structure and spectral features.^1^ One commonly adopted strategy is to employ relatively rigid ligand frameworks, such as those based on the twelve-membered ring macrocycles pyclen or cyclen (1,4,7,10-tetraazacyclododecane, 12-N_4_)^2–5^ and the nine-ring homologue 1,4,7-triazacyclononane, (9-N_3_).^6–9^ In these systems, the twist of the coordinated pendant arms around the lanthanide centre generates well-defined Δ or Λ chirality at the metal centre.^10–12^

Probing the chirality of the excited state of chiral lanthanide coordination complexes is made possible using circularly polarised luminescence (CPL) spectroscopy. CPL measures the differential emission of left- and right-circularly polarised light, providing key information on excited state structures of luminescent molecules. This technique is attracting significant interest in lanthanide chemistry for two reasons.^13–20^

First, lanthanide coordination complexes can be designed to be highly luminescent, with a high overall emission quantum yield (*ϕ*) as well as absorbing incident light strongly (high *ε*) *via* a sensitising chromophore, typically over the range 337 to 375 nm. When these chiral complexes also possess relatively large emission dissymmetry (*g*_em_) values, a strong CPL signal is generated.

Second, the CPL signature is often exceptionally sensitive to changes in the local coordination environment, notably through variations in overall complex symmetry, and as a result of perturbations of the ligand field through donor atom polarisability variation, both for the magnetic-dipole allowed transitions, *e.g.* Δ*J* = 1 for Eu(iii) complexes, and the electric-dipole allowed transitions, such as the hypersensitive Δ*J* = 2 and 4 manifolds. These properties have enabled lanthanide(III) complexes to emerge as powerful probes to report on the chirality of probe binding to biomolecules, like proteins or DNA, allowing detailed studies of binding stereoselectivity.^13,14^

Previous research by Neil *et al.* described a series of dynamically racemic *bis*-pyridylcarboxylate-9-N_3_ mono-cationic europium complexes, *i.e.*[EuL^1^]^2+^ and [EuL^2^]^+^ with the aim of signalling the presence of anionic targets including sialic acid, O–P-amino acids^21*a*^ phospho-peptides and lysophosphatidic acid (LPA).^22^ In particular, [EuL^1^]^2+^ was noted to display a selective induced circularly polarised luminescence (CPL) response to certain phosphorus(v) oxyanions.^21*a*^ This probe was designed with a methylammonium group at the *meta-*position of the *N*-benzyl moiety, doubling the overall complex positive charge. It was hypothesised at that time that the increase in positive charge strengthened the primary electrostatic interaction with target anions and, in some cases, the binding free energy could be further enhanced by directed hydrogen bonding between the ammonium group and, say, a metal-coordinated phosphate group.^23,24^ However, despite showing promising binding behaviour, the limited aqueous solubility of these complexes limited their utility for analytical applications in biofluids, as methanol had to be added to allow complex solution.

**Fig. 1:**
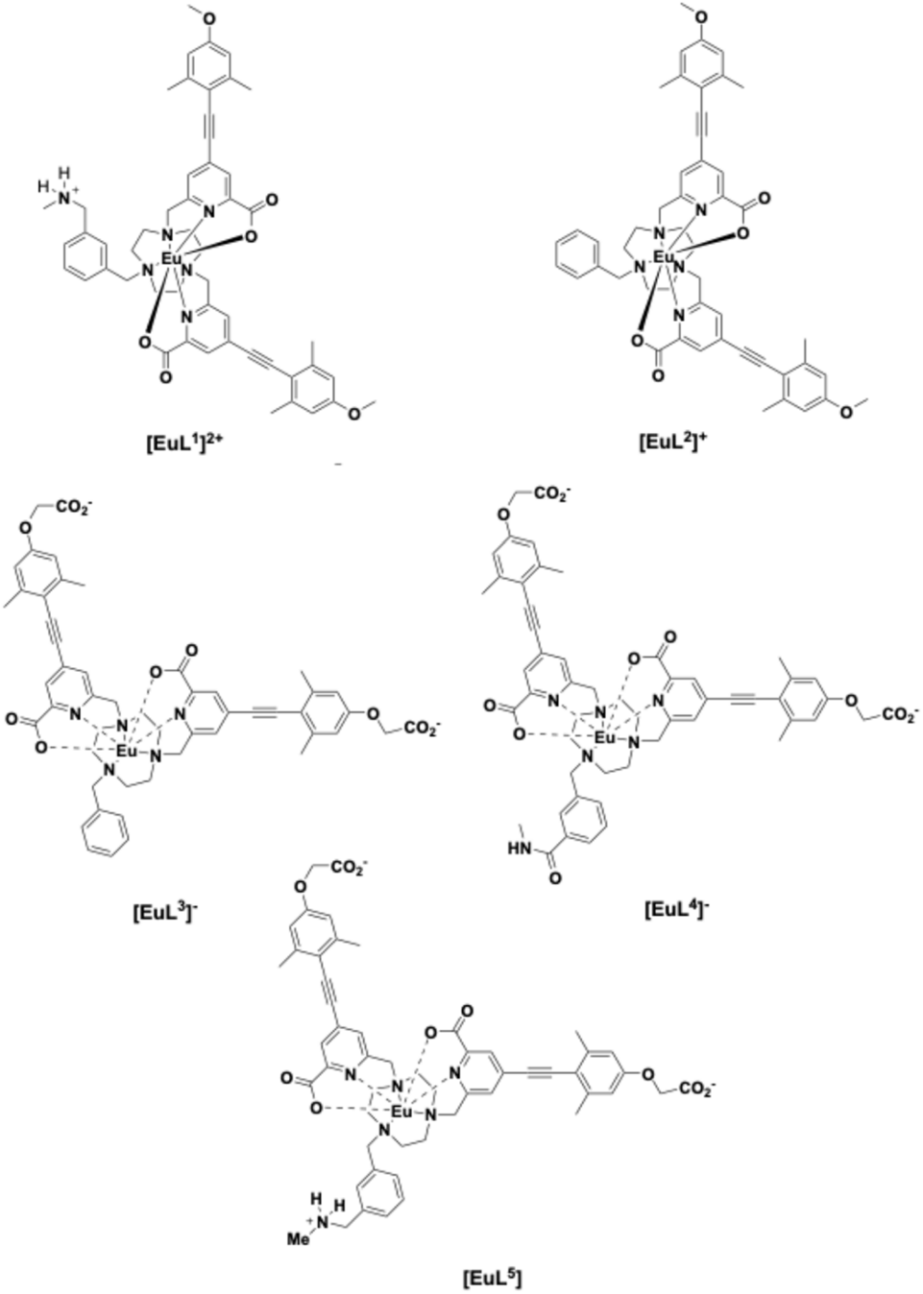
Structures of Eu complexes: [EuL^3–5^] are studied in this work.

With a view to improve aqueous solubility, we have introduced carboxylate groups at the distal positions of each aryl–alkyne chromophore, replacing the original methoxy substituents in [EuL^1^]^2+^. This modification was made in the hope that in aqueous solution, the carboxylic acid groups would be deprotonated, creating neutral or mono-anionic complexes for the complexes [EuL^3–5^]. The luminescence properties of each complex are reported here, focusing on a comparative analysis of binding behaviour with selected proteins and oxyanions, chosen where the related data exists with the prototype complexes, [EuL^1,2^]Fig. 1.

## Results and discussion

### Synthesis of the ligands and their Eu(iii) complexes

The antenna chromophore in each complex was synthesised *via* simple modifications to the methods reported by Neil *et al.* for the related *para*-methoxy analogues, [EuL^1,2^].^22^ The synthetic route began with formation of a 4-iodo pyridine diester derivative, proceeding *via* the more reactive *N*-acetyl pyridinium intermediate, followed by selective ester reduction with NaBH_4_ in ethanol to yield the alcohol, 1. In parallel, a TMS-protected terminal alkyne 2 was formed under palladium catalysis using Pd(PPh_3_)_4_ and CuI in Et_3_N, yielding the protected alkyne intermediate, 2 in 93% yield. Deprotection of the TMS group using triethylammonium fluoride proceeded efficiently to afford the deprotected derivative 3 in 45% yield. Finally, a second Sonogashira-coupling between 3 and 1 furnished the target chromophore 4.

**Fig. 2:**
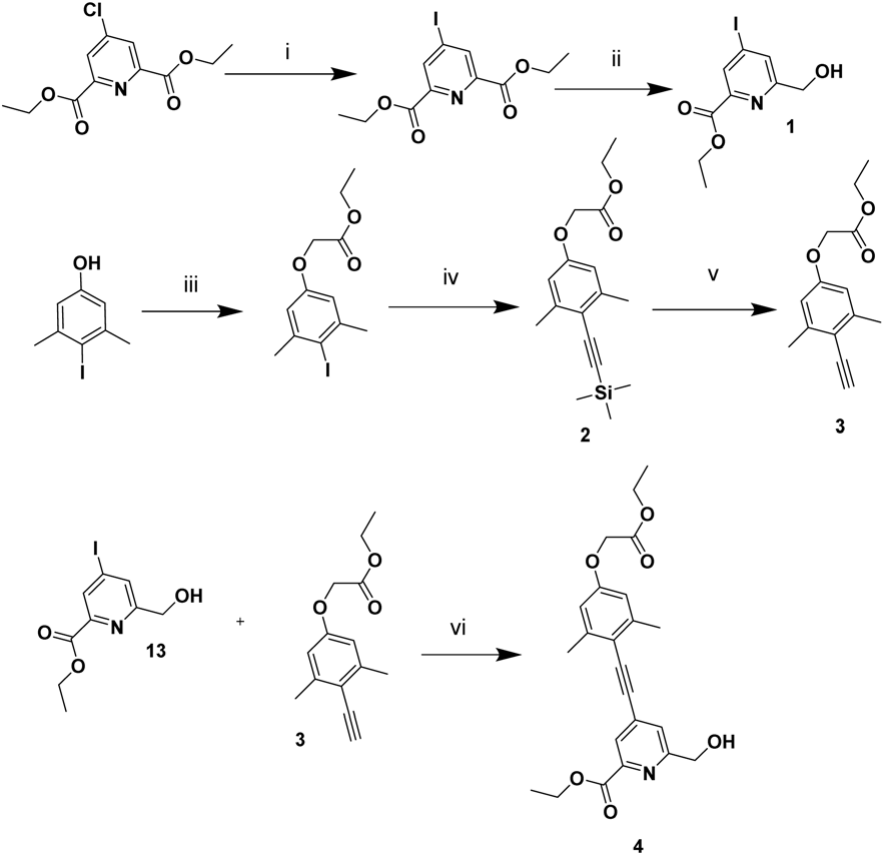
Synthetic route for intermediate, 4. (i) CH_3_COCl, NaI, MeCN, RT, 4 h, 86%; (ii) NaBH_4_, EtOH, 0 °C to 45 °C, 15 min, 50%; (iii) ethyl bromoacetate, K_2_CO_3_, acetone, reflux, 24 h, 98%; (iv) trimethylsilyl-ethyne, Pd(PPh_3_)_4_, CuI, Et_3_N, 55 °C, 48 h, 93%; (v) triethylammonium trihydrofluoride, THF, 30 °C, 72 h, 45%; (vi) Pd(dppf)Cl_2_, CuI, Et_3_N, 65 °C, 48 h, 70%. (Compound 13 should be changed to 1 in the figure).

**Fig. 3:**
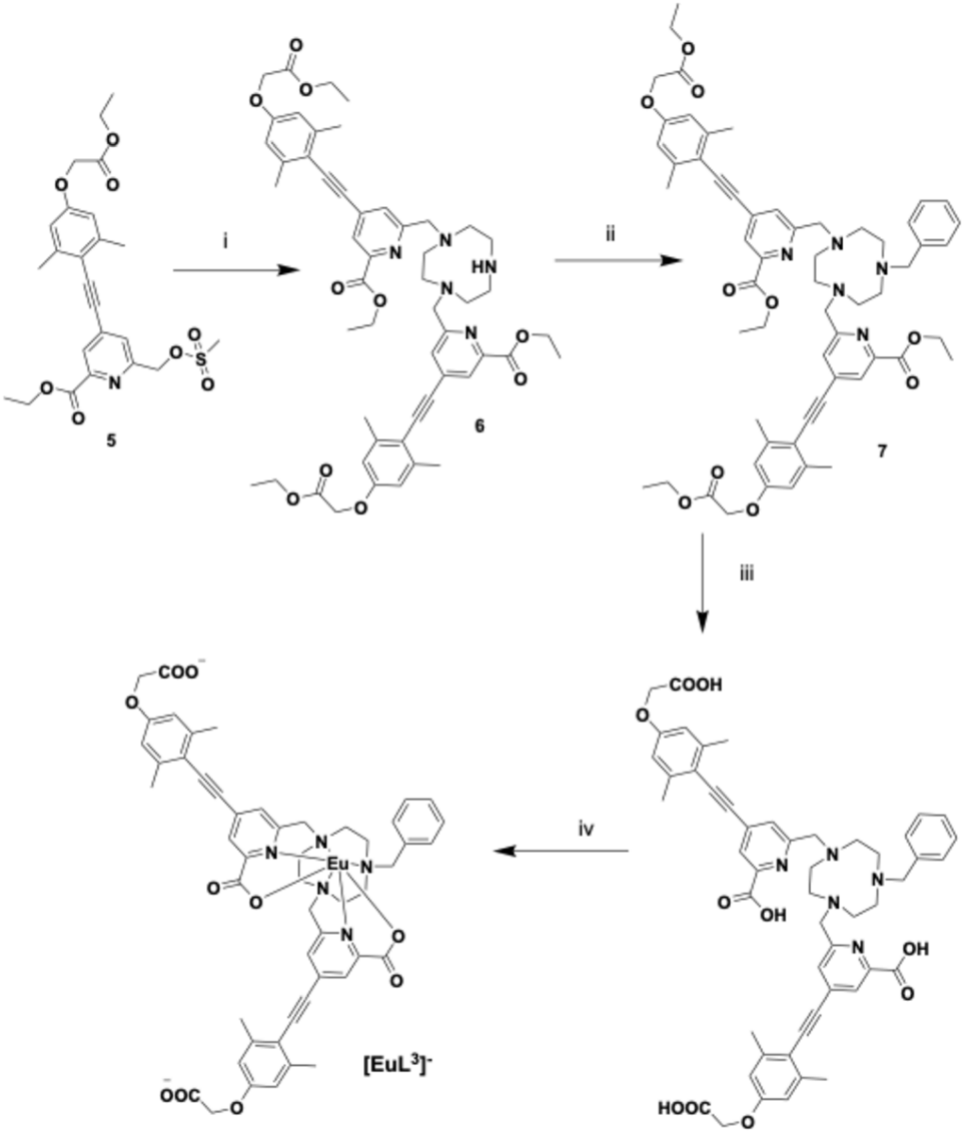
Synthesis of [EuL^3^]^−^: (i) 1,4,7-triazacyclononane trihydrochloride, K_2_CO_3_, MeCN, 60 °C, 2 h, 63%; (ii) benzyl bromide, K_2_CO_3_, 60 °C, 24 h, 84%; (iii) 0.4 M KOH, MeOH, H_2_O, RT, 95%; (iv) EuCl_3_^.^6H_2_O, pH 6.5, 65 °C, 24 h.

Each Eu complex in this work possesses the same antenna chromophore. Taking the synthesis of [EuL^3^]^−^ as an example of the strategy used (Fig. 2 and 3), direct dialkylation of 1,4,7-triazacyclononane with the mesylate 5 was performed first. To minimise formation of the tri-*N*-substituted derivative, the reaction was carried out using exactly two equivalents of the alkylating agent in relatively dilute solution (25 µmol mL^−1^). Under these conditions, the desired dialkylated product, 6, was obtained as the major product in 63% yield, following overnight reaction at 60 °C. The desired ligand was successfully isolated by preparative HPLC. Analysis of the crude reaction mixture by ESI-MS showed no evidence for the formation of the tri-alkylated by-product.

In the next step, the intermediate was reacted with benzyl bromide to afford compound 7 in 84% isolated yield (Fig. 3). Subsequent hydrolysis of the tri-ester 7 under basic conditions (0.4 M KOH in H_2_O/MeOH) furnished the carboxylic acid derivative, ligand L^3^, in high yield. Complexation of L^3^ with EuCl_3_ was carried out in methanol to yield the target complex [EuL3]^−^. Details of related syntheses of L^4^ and L^5^ and their Eu complexes are given in the SI.

### Photophysical properties of [EuL^3–5^]

**Fig. 4:**
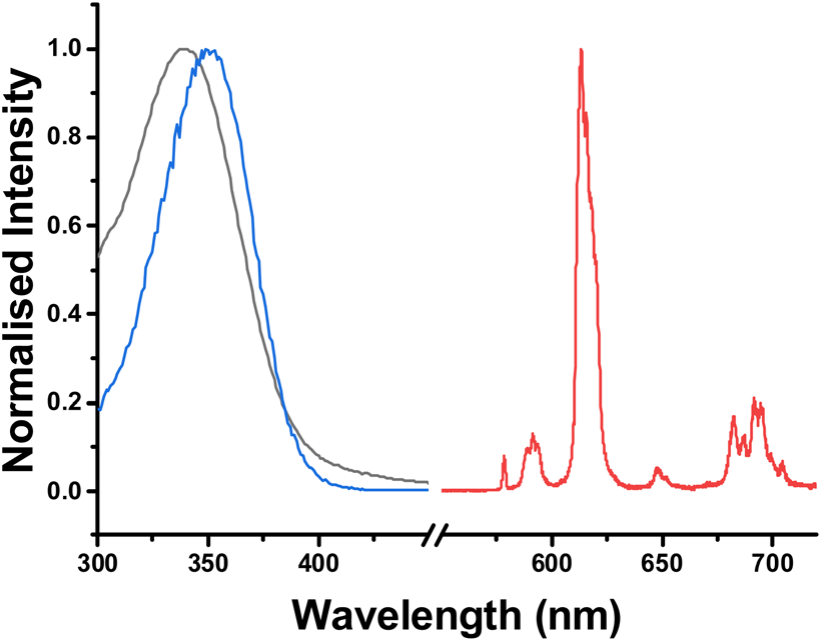
Absorption(black), excitation(blue) and emission spectra (red) for [EuL^4^]^−^ (DMSO : H_2_O 1 : 99, 298 K, *λ*_exc_ 350 nm): see Fig. S1 and S2 for very similar spectra with [EuL^3/5^].

Each complex in the series exhibits an absorption band at 280 nm; this band can be assigned to the π–π* transition of the local pyridine chromophore. In addition, every complex showed an intense absorption band between 346 and 348 nm (Table 1 and Fig. 4). This prominent feature originates from a strong ICT band within the ligand framework.^25,26^ The excitation and absorption spectra for each complex did not quite overlap. Given that the measurement was made for optically dilute samples with absorbance values of <0.05, such behaviour might be explained by the tendency of the ground state species to aggregate, as the excitation spectrum only shows absorption of a monomer.

**Table 1:** Selected photophysical properties of the Eu(iii) complexes a of L^3–5^ (298 K, DMSO : H_2_O 1 : 99; emission decay rate constants, *k* (equal to 1/*τ*) are the mean value of 3 separate measurements at 5 µM complex) concentration^a^

	[EuL^3^]^−^	[EuL^4^]^−^	[EuL^5^]
*λ*/nm	346	348	347
*ε*/mM^−1^ cm^−1^	34	35	34
*ϕ*	0.098	0.028	0.032
*k*(H_2_O)/ms^−1^	1.61(02)	1.66(01)	1.56(02)
*k*(D_2_O)/ms^−1^	1.38(01)	1.39(01)	1.33(01)
*Q*	0	0	0

a *q* values were determined using the semi-empirical equation for water solutions, *q* = 1.2[(*k*(H_2_O) − *k*(D_2_O) − 0.26).^27^

Heptadentate lanthanide complexes typically bind two water molecules in aqueous solution. However, for each of these Eu(iii) complexes, the measured *q* values^27^ were found to be close to zero, so that no water molecules are bound to the europium ion in its first coordination sphere. The lower water solubility of *N*-benzylated or *N*-methylated polyaza-macrocyclic ligands based on 9-N_3_ and 12-N_4_ has been noted earlier, *e.g.*, by Neil and Tripier *et al.*, respectively.^21^ With the series of complexes examined here, it is possible that a distal carboxylate group in a second molecule of complex coordinates to the metal ion causing oligomerisation, thereby reducing water solubility.

### Investigation of the binding behaviour of [EuL^3–5^]

The binding capabilities of the complexes [EuL^3–5^] were evaluated with a series of luminescence spectral titrations. Binding affinities toward various analytes, including bovine and human serum albumin, alpha-1-AGP, sodium bicarbonate, and a selective series of phosphorylated amino acids and peptides, were assessed, allowing data to be compared between these complexes and [EuL^1,2^].

For [EuL^3^]^−^ and [EuL^4^]^−^, owing to the insolubility in water caused by oligomerisation, stock solutions of each complex were prepared in DMSO and were diluted into the shown solvent, containing aqueous HEPES buffer. Results from binding studies of [EuL^3–5^] with bicarbonate and selected proteins, including the corresponding binding constants, log *K*, and minor variations in excited-state emission lifetimes, *τ*, are summarised in Table 2.

**Table 2:** Binding constant values, log *K*; excited state lifetimes, *τ*, for [EuL^3^]^−^, [EuL^4^]^−^, [EuL^5^] with added protein abd; (5 µM, *λ*_exc_ 350 nm, 10 mM HEPES, 298 K, pH 7.40)

	[EuL^3^]^−^a		[EuL^4^]^−^b		[EuL^5^]^b^	
	*τ*(ms)	log *K*	*τ*(ms)	log *K*	*τ*(ms)	log *K*
HSA	0.64(01)	6.68(05)	0.68(01)	6.41(03)	0.65(01)	c
BSA	0.64(01)	6.98(05)	0.64(02)	6.11(05)	0.67(01)	c
α_1_-AGP	c	c	0.67(01)	6.33(02)	0.68(02)	5.84(06)
NaHCO_3_	0.66(01)	4.98(07)	0.69(01)	3.21(03)	0.68(01)	3.15(05)

a For [EuL^3^]^−^ due to solubility issues: 33% buffer/66%MeOH.

b 99% Buffer/1% DMSO; with [EuL^5^] using a 2 : 1 MeOH/HEPES buffer, similar log *K* values were measured.

c The complex binding behaviour observed could not be fitted satisfactorily to a 1 : 1 binding isotherm (Fig. S7).

d Values in parenthesis refer to experimental errors associated with three independent determinations.

### Binding behaviour of the Eu complexes in buffered solution

[EuL^4^]^−^ and [EuL^5^] were dissolved in the minimum volume of dimethyl sulfoxide (DMSO) to prepare stock solutions for subsequent experiments, so that the medium of analysis was 1 : 99 (DMSO/aqueous buffer). The greater insolubility of [EuL^3^]^−^ in water meant that its binding behaviour was assessed in 2 : 1 MeOH/buffer, but as such a mixed solvent may lead to protein denaturation these binding affinities cannot be directly compared.

**Fig. 5:**
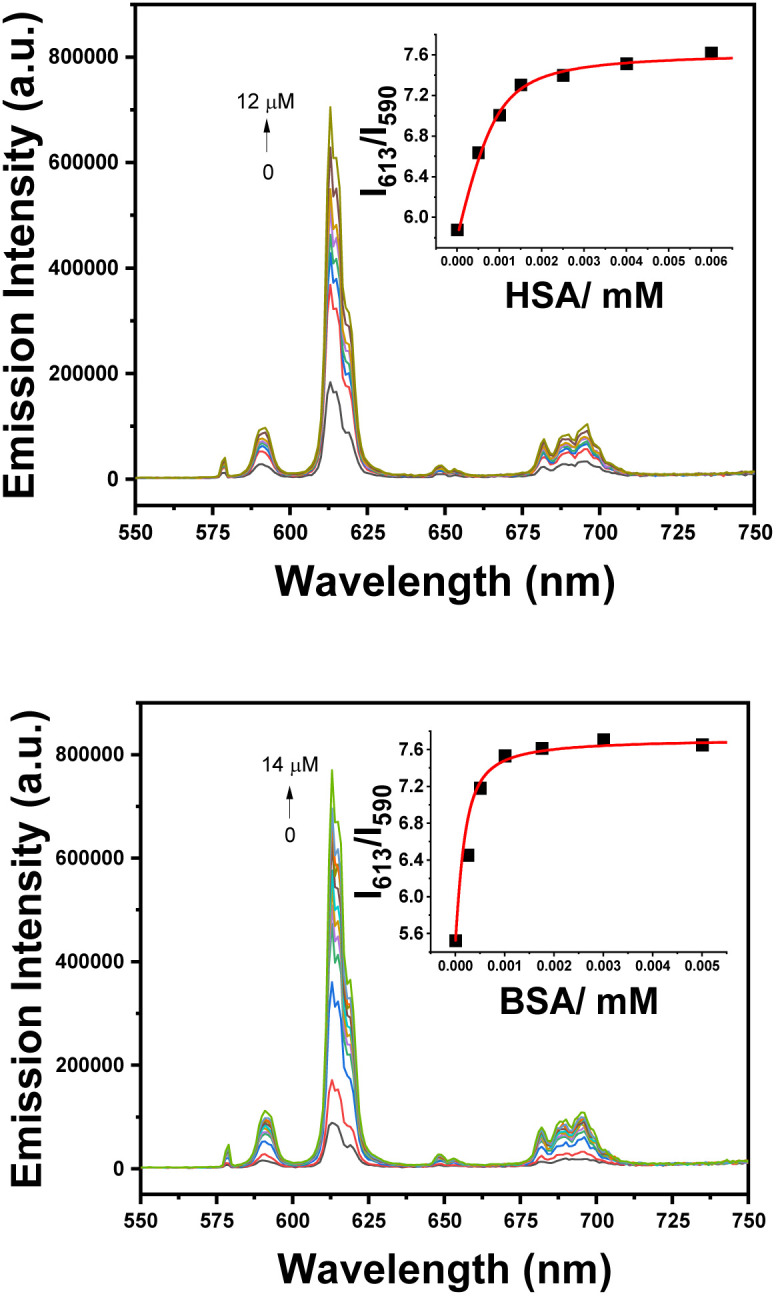
Variation of the total emission intensity and the Δ*J* = 2/Δ*J* = 1 ratio on addition of HSA (log *K* = 6.68 (05)) and BSA (log *K* = 6.98 (05)) to [EuL^3^]^−^; ([EuL^3^]^−^ 5 µM, the inset assumes 1 : 1 binding stoichiometry (MeOH : H_2_O 2 : 1, 298 K, *λ*_exc_ 350 nm).

With [EuL^3^]^−^, significant enhancements were observed both in overall luminescence intensity and the intensity ratio of the Δ*J* = 2/Δ*J* = 1 transitions, following addition of HSA or BSA to the [EuL^3^]^−^ solution (Fig. 5).

**Fig. 6:**
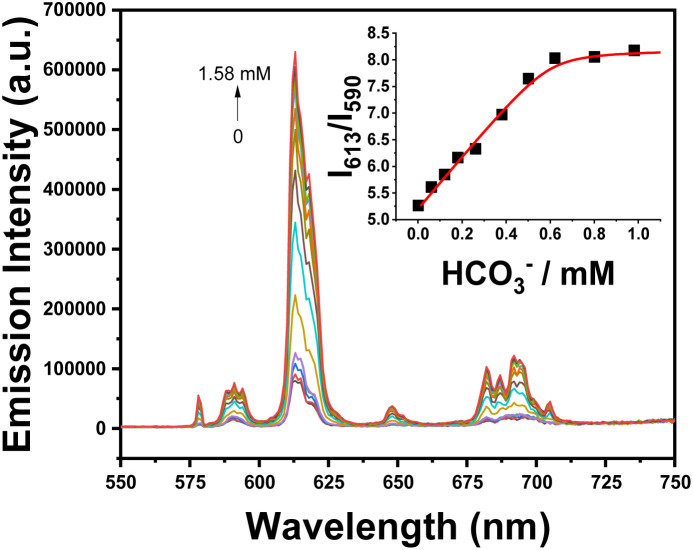
Change in Eu emission spectra upon addition of bicarbonate to [EuL^3^]^−^; ([EuL^3^]^−^ 5 µM, the inset assumes a 1 : 1 binding isotherm (log *K* = 4.98 (03)), MeOH : H_2_O 2 : 1, 298 K, *λ*_exc_ 350 nm).

With added bicarbonate (Fig. 6), the large increase in total emission intensity was associated with the binding of the two polarisable oxygen atoms of carbonate to the Eu(iii) centre.^28,29^ This chelating interaction may promote the dissociation of the aggregated complex and led to an enhancement of the luminescence intensity by a factor of 15 (Fig. 6). The apparent binding constant (Table 2) for [EuL^3^]^−^ with bicarbonate was deduced by examining the variation of the Δ*J* = 2/Δ*J* = 1 emission intensity ratio as a function of added salt and was found to be log *K* = 4.98, fitting to a 1 : 1 binding model, using iterative non-linear, least-squares analysis. The rather high apparent affinity for bicarbonate is notable, as most structurally similar Eu(iii) and Gd(iii) complexes have an affinity in water in the range log *K* 2.5 to 3.5.^30^ However, in the mixed MeOH/H_2_O solvent system used here, the carbonate and bicarbonate anion solvation energies are likely to much lower than in pure water, and specific solvation of the complex chromophore by MeOH may occur as well, so that log *K* values in the mixed and pure solvent systems should not be directly compared.

Following addition of phosphorylated amino acids (O–P-Ser/Thr/Tyr) or selected phosphorylated peptides (GAPY·KF, GS·PFKF, GS·PY·KF), only a slight decrease in luminescence emission intensity was observed, with no accompanying changes in spectral form. Indeed, with these *O*-phospho-anions, no significant evidence for binding was observed with [EuL^3^]^−^. While minor spectral changes were detected following addition of serum albumin, the methanol-dominated solvent system proved unsuitable for maintaining protein stability and its native conformation. Consequently, the experiments with [EuL^3^]^−^ serve only as a control here, and no further binding studies were performed with it.

The binding behaviour of [EuL^4^]^−^ with added proteins, phosphorylated amino acids, and anions was investigated next. Following the addition of *O*-phosphorylated tyrosine/serine to [EuL^4^]^−^ a decrease in total emission intensity was observed, whereas no significant changes in emission intensity or spectral form were noted following addition of *O*-phosphorylated threonine. Similar trends were observed in the variation of total emission intensity for [EuL^4^]^−^ when adding HSA, BSA, α_1_-AGP or bicarbonate (Fig. S3–S6 and S9): with added protein and bicarbonate the overall emission intensity increased by factors of 4 to 8, and binding constants could be estimated assuming 1 : 1 stoichiometry (Table 2).

Binding experiments were also conducted with selected phosphorylated peptides. The introduction of hexapeptides containing a single phosphorylated serine or tyrosine residue (GS·PFKF, GAPY·KF) did not induce any measurable change in either the luminescence intensity or spectral form of [EuL^5^] (Fig. S6). In contrast, titration with the doubly phosphorylated peptide GS·PY·KF resulted in a detectable increase in Eu(iii) emission intensity. Ratiometric analysis examining changes in the Δ*J* = 2/Δ*J* = 1 intensity ratio further confirmed this distinct response (Fig. S6), showing a clear trend different from the near-zero response observed with the singly phosphorylated peptides. This behaviour suggests that the presence of two phosphate groups may provide an electrostatic advantage, enabling stronger binding to the Eu(iii) centre.

The complex [EuL^5^] was designed with a proximate charged ammonium group (CH_2_NH_2_Me^+^) on the benzyl ring, with the idea of enhancing affinity by introducing an additional local Coulombic and/or hydrogen bonding interaction.^22^ The behaviour of [EuL^5^] toward protein and phosphorylated amino acids and peptides was investigated by monitoring changes in both total emission and circularly polarised luminescence, as well as by monitoring changes in excited state lifetimes. Following the addition of human or bovine serum albumin, the emission spectral intensity increased but the spectral fingerprint and intensity ratios did not change. Furthermore, compared to the behaviour with HSA, total intensity changes caused by adding BSA were minimal, (Fig. S7).

Binding of [EuL^4^]^−^ and [EuL^5^] to α_1_-AGP was also compared. The addition of α_1_-AGP enhanced the luminescence intensity of each complex. However, the enhancement observed with [EuL^4^]^−^ (Fig. S5) was significantly more pronounced than with [EuL^5^] (Fig. S8). The corresponding binding constants were log *K* = 6.33 for [EuL^4^]^−^ and log *K* = 5.84 for [EuL^5^] (Table 2). With [EuL^5^] upon addition of various phosphorylated species (Fig. S10 and S11), a decrease of luminescence intensity of 50% was observed.

### CPL studies of protein binding and complex speciation

CPL spectroscopy has been increasingly used recently to provide more detailed information on the absolute configuration of the primary emissive species and to give better spectral resolution, as neighbouring transitions of opposite sign within a given manifold can be clearly distinguished.^11,13–20^

**Fig. 7:**
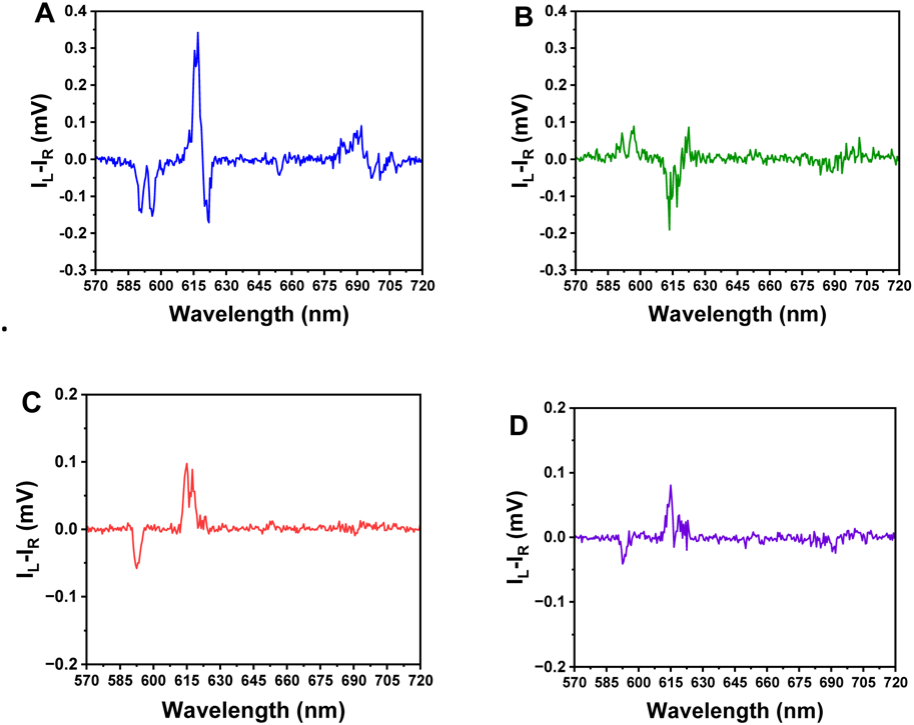
CPL spectra of [EuL^4^]^−^ in the presence of a two-fold excess of HSA (A) or BSA (B), and a six-fold excess of GSPYKF (C), GS·PY·KF (D). (*λ*_εxc_ 340 nm, 298 K; 5 µM complex; 1 : 99 (DMSO/water). In the absence of added protein or peptide no detectable CPL was observed, as the complex is dynamically racemic.

CPL spectra for [EuL^4^]^−^ were acquired in the presence of HSA, BSA, and selected phosphorylated peptides. Preliminary studies monitoring the total Eu(iii) emission intensity and spectral profile had shown only a modest signal enhancement in the presence of serum albumin. In contrast, addition of phosphorylated amino acids and peptides generally induced quenching of the total emission intensity, without changing the spectral fingerprint. Attempts to measure CPL profiles for [EuL^4^]^−^ in the presence of either BSA (Fig. 7) and selected phosphorylated peptides yielded spectra of low S/N and poor quality. With added HSA, however, a higher S/N CPL spectrum was obtained, and a relatively strong CPL signal was found, associated with the magnetic dipole-allowed ^5^D_0_/^7^F_1_ and electric dipole-allowed ^5^D_0_/^7^F_2_ transitions (*g*_em_ = −0.06 (590.5); −0.04 (596): +0.01 (616)).

Notwithstanding the rather weak signal observed, comparing the CPL spectra of [EuL^4^]^−^ with added HSA and BSA, the protein bound complexes show oppositely signed CPL signatures, as seen in the Δ*J* = 1 and Δ*J* = 2 transitions around 590 and 620 nm, respectively (Fig. 7). The induced CPL spectra of these adducts were compared with the spectral signatures of related, enantiopure C_3_-symmetric complexes, for which well-defined sign sequences for the main transitions in the Δ*J* = 1 and Δ*J* = 2 manifolds have been repeatedly observed for a variety of systems with C_1_ and C_3_ symmetry.^10,21,22,31^ The absolute configuration and precise structure of these reference complexes had been unambiguously determined by X-ray crystallography^31,32^ which allows the assignment of the HSA-bound adduct to a Λ stereochemical configuration at the metal centre, with a δδδ conformation across the three Eu–N–C–C–N chelate rings associated with the 9-membered macrocycle.^10,11^ This unexpected, albeit empirical, finding demonstrates again that CPL spectroscopy is a powerful technique to distinguish the absolute configuration of lanthanide ternary complex adducts.^13^

**Fig. 8:**
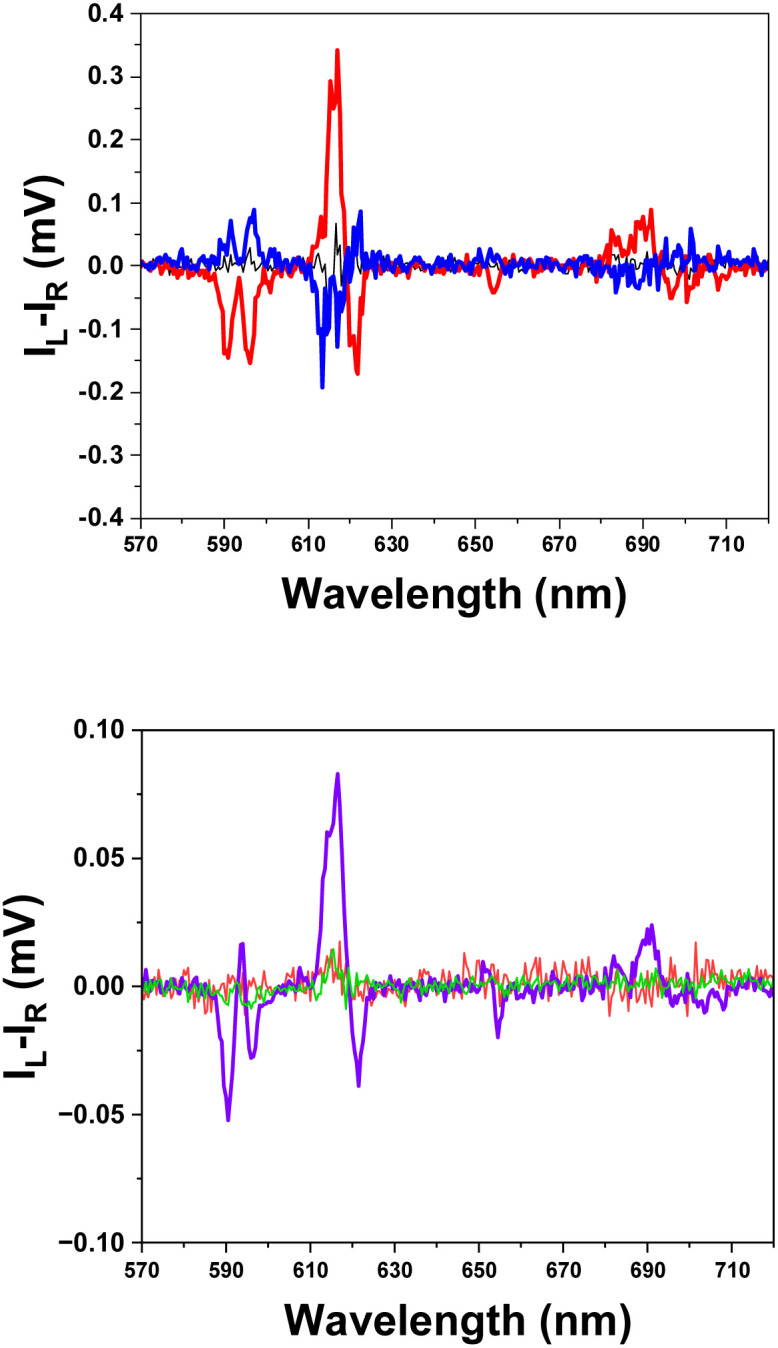
(Upper): CPL spectra of free [EuL^4^]^−^ (black) and when in the presence of HSA (red) and BSA (blue), *λ*_exc_ 350 nm, H_2_O, 298 K, ([EuL^4^]^−^ 5 µM, serum albumin 10 µM; (lower): CPL spectra of [EuL^4^]^−^ dissolved in 0.05 mM bicarbonate (red) in the presence of HSA (purple) or BSA (green); (*λ*_exc_ 350 nm, H_2_O, 298 K, ([EuL^4^]^−^ 5 µM, serum albumin 10 µM. 0.05 mM NaHCO_3_).

Given the high physiological concentration of bicarbonate, (*e.g.* 23 mM in human serum) its potential to interfere with albumin-lanthanide complex binding was investigated. Specifically, we sought to determine whether the presence of bicarbonate influences the CPL signal of the protein-bound adducts. A control experiment confirmed that [EuL^4^]^−^, in the presence of 0.05 mM bicarbonate only, gave rise to no detectable CPL signal (Fig. 8). Similarly, the characteristic CPL signal, indicative of a Δ-configuration protein-adduct with [EuL^4^]^−^ in the presence of BSA, was fully quenched upon addition of bicarbonate (Fig. 8 green). Such behaviour suggested that [EuL^4^]^−^ binds more strongly to hydrogencarbonate than BSA, disrupting BSA-[EuL^4^]^−^ adduct formation. Surprisingly, the strong CPL signal persisted when HSA was added to [EuL^4^]^−^, *i.e.* even in the presence of competing bicarbonate ions (Fig. 8 purple). This result strongly suggests that the binding affinity of [EuL^4^]^−^ for HSA is significantly stronger than for bicarbonate, and the chiral Λ adduct is formed, notwithstanding the presence of excess anion.

Additional control experiments were undertaken to allow a comparison of the Eu spectral fingerprints and to assess ternary complex speciation using electrospray ionisation mass spectroscopy. With [EuL^4^]^−^, a set of LC-ESMS analyses was undertaken, with three different mobile phase compositions: 0.1% aqueous formic acid/MeCN and then, replacing the formic acid by 10 mM NH_4_OAc or 10 mM ammonium bicarbonate solution. Acetate and carbonate have previously been established by X-ray crystallography and solution NMR studies to chelate to Eu, displacing bound water or weakly coordinated ligand donors.^24,28,33,34^

**Fig. 9:**
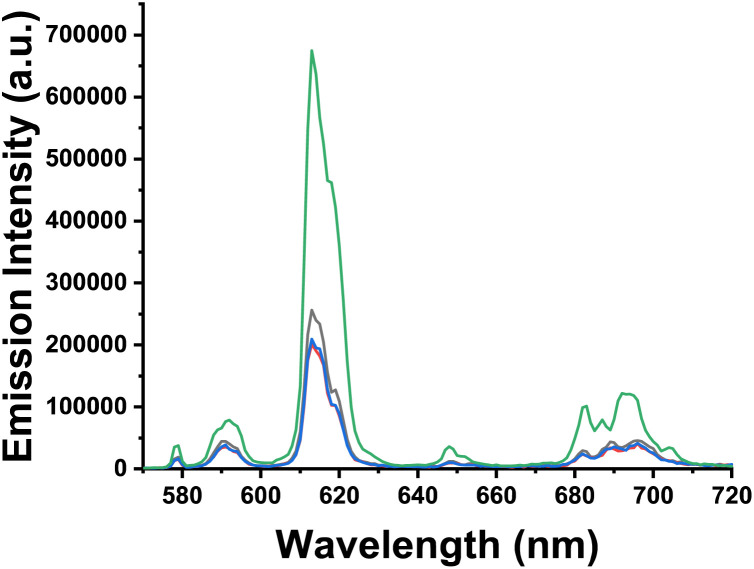
Emission spectra for [EuL^4^]^−^ (black: water pH 7.4; red: in 2% aqueous DMSO; blue: 12 mM aq. ammonium acetate solution; green: 10 mM aq. ammonium carbonate solution, 298 K, *λ*_exc_ 350 nm).

Total emission spectra for [EuL^4^]^−^ were compared in pure water, in water plus 2% DMSO, and then following additions of ammonium salts to make aqueous solutions 12 mM in ammonium acetate or 10 mM in ammonium carbonate (Fig. 9). The only significant spectral change occurred with added carbonate, when the Δ*J* = 2/Δ*J* = 1 intensity ratio increased by 50%. Such behaviour is in accord with Eu spectral changes observed for many europium or terbium-carbonate adducts.^20,21^ The absence of change with added DMSO or acetate is consistent with retention of a common Eu coordination sphere in the primary emissive species, *i.e.* the presence of a europium-coordinated carboxylate for the minimally soluble complex on its own (*via* intermolecular carboxylate ligation) and in its monomeric adduct with a chelating acetate ion.

A series of LC-ESMS analyses lent support to this hypothesis: with added formic acid and [EuL^4^]^−^, a singly charged formate adduct was observed in negative ion ESMS, with the two major peaks at 1143/1145 ([(M–H) + HCO_2_]^−^ for the 151-Eu and 153-Eu isotopic species. When 10 mM acetate was present, the corresponding isotope pattern appeared at 1157/1159 [(M–H) + CH_3_CO_2_]^−^, but with bicarbonate present, major peaks were observed at 1116 and 1118 [(M–2H) + OH]^−^. With added bicarbonate the hydroxide adduct is observed rather than the carbonate adduct, presumably as a result of a higher local proton concentration in the electrospray chamber. Calculated and experimental isotope patterns showed good agreement, for each anion adduct observed (Fig. S18 and S19).

Recently, in a cyclen-based chiral Eu complex with a structurally similar arylalkynl-pyridyl chromophore (albeit lacking the pyridine-6-carboxylate), it was shown that the Eu complex bound much more strongly to HSA compared to BSA.^11^ The Δ-Eu cyclen complex bound selectively to drug-site 1 with each serum albumin. With HSA, additional chelation of the side chain carboxylate of Glu-188 to Eu occurred, displacing coordinated water.^11^ This residue is absent in BSA and explained the pronounced selectivity in binding. However, here the complexes are based on triazacyclononane and the complex has a different shape and coordination environment. Evidently, the two situations cannot simply be compared.

Overall, these results demonstrate that [EuL^4^]^−^ binds to human and bovine serum albumin with a similar affinity, but the metal complex seems to adopt a different absolute configuration in each diastereomeric adduct. These differences were highlighted using CPL spectroscopy, notably in the absence and presence of added bicarbonate.

## Conclusions and summary

Three racemic Eu(iii) complexes [Eu^3–5^]^−^ based on the triazacyclononane framework have been synthesised and their solution binding behaviour compared. Among these, only [EuL^4^]^−^ demonstrated a very different CPL profile for human compared to bovine serum albumin. This was evidenced by the oppositely signed CPL signals observed in their respective adducts. The binding of [EuL^4^]^−^ to HSA persisted even in the presence of excess competing bicarbonate. CPL spectroscopy clearly indicated that the resulting protein-bound adduct adopted a Λ stereochemical configuration at the metal centre. Disappointingly, each of the three complexes still has poor water solubility, which is hypothesised to relate to their tendency to oligomerise in aqueous media.

To support the hypothesis of complex self-aggregation, bicarbonate was used as a competing agent, as the very low water solubility of the free complex precluded the use of NMR DOSY experiments. Upon introducing bicarbonate into an aqueous suspension of the complex (20 µM), the solid completely dissolved. We hypothesise that enhancement of solubility occurs because the metal centre of the complex undergoes bidentate coordination with the bicarbonate ion, replacing the coordination of the carboxylate group, creating a more negatively charged species with enhanced water solubility. This behaviour was found to be reversible; upon acidification of the solution (back to pH 4), the bicarbonate ion dissociated from the metal centre, CO_2_ evolved, and the complex slowly reprecipitated. The large increase in Eu emission intensity in the carbonate adduct may also be explained by the fact that the aggregated complex is less emissive than the monomeric carbonate ternary adduct.

Taken together, these observations suggest that the series of complexes containing two peripheral carboxylate groups can engage in metal-centred intermolecular coordination, which drives their aggregation and limits solubility in pure water. However, under physiological conditions, the high ambient concentration of bicarbonate naturally favours the solubilised, monomeric carbonate adduct. Consequently, despite their poor intrinsic aqueous solubility, these complexes show promise as the basis for development. Furthermore, the use of peripheral sulphonate groups in the ligand, rather than using carboxylate groups to enhance water solubility might avert the intermolecular aggregation tendency.

## Author contributions

The project was conceived by DP, and the manuscript was written by DP and HL; HL carried out the synthesis and characterisation work, the measurements of binding constants, rate constants and lifetimes. DJB recorded the CPL spectra under direction from RP.

## Conflicts of interest

There are no conflicts to declare.

## Supplementary Material

RA-016-D6RA02760A-s001

## Data Availability

The additional experimental data associated with this article have been included in the supplementary information (SI) section. Any additional data or raw data files for spectral titrations can be obtained by request to the corresponding author. Supplementary information: methods and materials, synthesis and characterisation, CPL and emission spectroscopy and binding studies. See DOI: https://doi.org/10.1039/d6ra02760a.
