# Supplementary material for: Protein binding regulates complex configuration: comparative analysis of three dynamically racemic europium(iii) complexes
Source: RSC Adv. 2026 May 18;16(28):26165–72. doi: 10.1039/d6ra02760a (PMC13181362; doi:10.1039/d6ra02760a)
Supplement: RA-016-D6RA02760A-s001 [file RA-016-D6RA02760A-s001.pdf]

Supplementary Information (SI) for RSC Advances.
This journal is © The Royal Society of Chemistry 2026

Supplementary Information

Protein binding regulates complex configuration: comparative analysis of three dynamically racemic europium(III) complexes

Huishan Li, [a] Dominic J. Black, [b] Robert Pal [b] and David Parker* [a]

[a] Department of Chemistry, Hong Kong Baptist University, Kowloon Tong, Hong Kong

[b] Department of Chemistry, Durham University, South Road, Durham DH1 3LE, UK

General Experimental: Reagents and Methods

Reagents. All reagents were used without further purification as purchased. $EuCl_3.6H_2O$ was purchased from Macklin and Sigma-Aldrich (all 99.99% trace metal bases). Solvents were laboratory grade and were dried over appropriate drying agents when necessary. Air sensitive reactions were carried out under nitrogen using Schlenk-line techniques.

Chromatography. Thin layer chromatography was carried out on silica plates (Merck TLC Silica gel 60 F_{254}) and visualized under UV irradiation (254/365 nm). Preparative column chromatography was performed using Biotage® Isolera™ One equipped with 200-800 nm UV-Vis detector and Biotage® Rening Cartridge – High Performance 40-63 µm. Crudes were loaded on silica gel (Bidepharm, 100-200 mesh).

Melting point measurement. Melting points were recorded using a Cole-Parmer® MP-250D-F apparatus and are uncorrected.

Nuclear magnetic resonance spectroscopy. NMR spectra were recorded on a Bruker Ultrashield 400 Plus NMR spectrometer (^{1}H NMR on 400 MHz) at 296 K. The ^{1}H NMR chemical shifts were referenced to the corresponding solvent peak (7.26 for $CDCl_3$, 3.31 for CD_3OD). The following abbreviations were used to explain the multiplicities: s = singlet, d = doublet, t = triplet, dt = doublet of triplet, q = quartet, m = multiplet.

Mass spectrometry. Low-resolution mass spectra, reported as m/z, were conducted using a SCIEX 3200Q ESI mass spectrometer for monitoring reaction and determining collected fraction during purification of product. High-resolution mass spectra were obtained from a Bruker Autoflex MALDI-TOF mass spectrometer.

HPLC analysis. HPLC analyses and purifications were performed at 296 K. All chromatograms were reported monitoring absorbance at 254nm and 330nm. Semi-preparative High

Performance Liquid Chromatograph (Shimadzu) LC-20AR, LC-20AR Solvent Delivery Pump, DGU-40 Degassing unit, LH-40 Liquid Handler, SPD-M40 Photodiode Array Detector, FRC-40 Fraction Collector, CBM-40 System Controller and XBridge® Prep C18 OBD™ column (5 µm, 19 × 100 mm).

Various chromatographic systems were used for analytical and preparative HPLC:

Method A: flow rate 5.0 mL/min with H_2O (0.1% TFA) – 20% MeCN (0.1% TFA) as eluents (linear gradient to 44% MeCN (0.1% TFA) [20 min].

Method B: flow rate 5.0 mL/min with H_2O (0.1% TFA) – 20% MeCN (0.1% TFA) as eluents (linear gradient to 41% MeCN (0.1% TFA) [25 min].

Method C: flow rate 5.0 mL/min with H_2O (0.1% TFA) – 20% MeCN (0.1% TFA) as eluents (linear gradient to 49% MeCN (0.1% TFA) [30 min].

Method D: flow rate 5.0 mL/min with H_2O (0.1% TFA) – 20% MeCN (0.1% TFA) as eluents (linear gradient to 60% MeCN (0.1% TFA) [40 min].

Method E: flow rate 5.0 mL/min with H_2O (0.1% TFA) – 45% MeCN (0.1% TFA) as eluents (linear gradient to 80% MeCN (0.1% TFA) [40 min].

Photophysical measurements. The ultraviolet-visible absorption spectra were measured in the range of 200-800 nm using an Agilent Technologies Cary 8454 UV-Vis spectrophotometer. The emission and excitation spectra were recorded using a Horiba FluoroMax®-4 instrument equipped with a 450 W xenon lamp operating FluorEssence (v3.8) software. Lifetime measurements were recorded using a Horiba Fluorolog®-3 instrument equipped with a 355 nm spectra-LED pulsed light source using DataStation (v2.7) software.

CPL spectroscopy

PEM-CPL spectrometer[12]: CPL was measured with a home-built (modular) spectrometer. The excitation source was a broad band (200 – 1000 nm) laser- driven light source EQ 99 (Elliot Scientific). The excitation wavelength was selected by feeding the broadband light into an Acton SP-2155 monochromator (Princeton Instruments); the collimated light was focused into the sample cell (1 cm quartz cuvette). Sample PL emission was collected perpendicular to the excitation direction with a lens (f = 150 mm). The emission was fed through a photo-elastic modulator (PEM) (Hinds Series II/FS42AA) and through a linear sheet polariser (Comar). The light was then focused into a second scanning monochromator (Acton SP-2155) and subsequently on to a photomultiplier tube (PMT) (Hamamatsu H10723 series). The detection of the CPL signal was achieved using the field modulation lock-in technique. The electronic signal from the PMT was fed into a lock-in amplifier (Hinds Instruments Signaloc Model 2100). The reference signal for the lock-in detection was

provided by the PEM control unit. The monchromators, PEM control unit and lock-in amplifier were interfaced to a desktop PC and controlled by a custom-written LabView graphic user interface. The lock-in amplifier provided two signals, an AC signal corresponding to (I_L- I_R) and a DC signal corresponding to (I_L + I_R) after background subtraction. The emission dissymmetry factor was therefore readily obtained from the experimental data, as 2 AC/DC.

Spectral calibration of the scanning monochromator was performed using a Hg-Ar calibration lamp (Ocean Optics). A correction factor for the wavelength dependence of the detection system was constructed using a calibrated lamp (Ocean Optics). The measured raw data was subsequently corrected using this correction factor. The validation of the CPL detection systems was achieved using light emitting diodes (LEDs) at various emission wavelengths. The LED was mounted in the sample holder and the light from the LED was fed through a broad band polarising filter and $\lambda/4$ plate (Ocean Optics) to generate circularly polarised light. Prior to all measurements, the $\lambda/4$ plate and a LED were used to set the phase of the lock-in amplifier correctly. The emission spectra were recorded with 0.5 nm step size and the slits of the detection monochromator were set to a slit width corresponding to a spectral resolution of 0.25 nm. CPL spectra (as well as total emission spectra) were obtained through an averaging procedure of several scans. The CPL spectra were smoothed using a shape-preserving Savitzky-Golay smoothing (polynomial order 5, window size 9 with reflection at the boundaries) to reduce the influence of noise and enhance visual appearance; all calculations were carried out using raw spectral data. Analysis of smoothed vs raw data was used to help to estimate the uncertainty in the stated gem factors, which was typically ±10%.

Binding constant determinations. Each Eu(III) complex was prepared as a 1 mM solution. A 1 mM stock solution of each analyte was freshly prepared in deionized water. Titrations were performed by carefully adding incremental volumes of the analyte stock to the complex solution using an automatic pipette, ensuring minimal agitation. The total added volume was always maintained at $\leq$ 5% of the initial sample volume to avoid significant dilution effects.

The binding interaction was monitored by plotting the intensity of the hypersensitive Eu^{3+} transition at 614 nm against the analyte concentration. All measurements were performed in triplicate. The apparent binding constant (*K*) for each complex was calculated using a 1:1 binding model according to the following equation:

$$u + X \leftrightarrow EuX$$

$$[X] = \frac{\frac{(F - F_0)/(F_1 - F_0)}{K} + [Eu] \times \frac{F - F_0}{F_1 - F_0} - [Eu] \times (\frac{F - F_0}{F_1 - F_0})^2}{1 - (F - F_0)/(F_1 - F_{0)}}$$

$[X]$: the total concentration of protein in the solution

$[Eu]$: the total concentration of the complex

K: the binding constant

F: the ratio/intensity of selected peaks

F_0: the initial ratio/intensity of selected peaks

F_1: the final ratio/intensity of selected peaks

$[EuX]$: the concentration of the appropriate BSA or drug-coordinated complex

$[Xf]$: the concentration of free BSA or drug in the mixture

$[Euf]$: the concentration of the free complex

Synthetic Procedures for [EuL$^{3-5}$] and precursors

Compounds **1** to **5** have been reported in the literature[1] and were made by minor modifications to the reported methods.

Diethyl 6,6'-((1,4,7-triazacyclononane-1,4-diyl)bis(methylene))bis(4-((4-(2-ethoxy-2-oxoethoxy)-2,6-dimethylphenyl)ethynyl)picolinate), 6

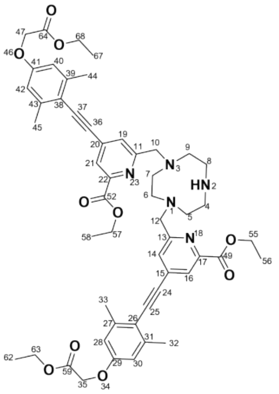

1,4,7-Triazacyclononane trihydrochloride (24 mg, 0.18 mmol) and the mesylate **5** (180 mg, 0.36 mmol) were dissolved in anhydrous CH_3CN (30 mL) and K_2CO_3 (124 mg, 0.92 mmol) was added. The mixture was stirred under nitrogen at 60 °C and carefully monitored by LC-MS. After 2 h significant formation on the bis-alkylated product was observed and the reaction was cooled and filtered to remove excess potassium salts. The solvent was removed under reduced pressure and the crude material was purified by HPLC (*Method D*: ***t$_R$:*** 19.4 min) to give a yellow glassy solid (56 mg). **^{1}H NMR** (296 K, 400 MHz, Chloroform-*d*) δ 7.86 (s, 2H, $H^{16,21}$), 7.46 (s, 2H, $H^{14,19}$), 6.64 (s, 4H, $H^{28,30,40,42}$), 4.62 (s, 4H, $H^{35,47}$), 4.52 (s, br. 4H, $H^{10,12}$), 4.28 (m, *J* 7.0, 8H, $H^{55,57,63,68}$), 3.75 (m. br. 8H, $H^{5,6,7,9}$), 3.57 (s, 4H, $H^{4,8}$), 2.46 (s, 12H, $H^{32,33,44,45}$), 1.30 (m, *J* 7.0, 12H, $H^{56,58,62,67}$). **^{13}C NMR** (296 K, 101 MHz, Chloroform-*d*) δ 168.7

($C^{59,64}$), 164.2 ($C^{49,52}$), 158.6 ($C^{29,41}$), 155.8 ($C^{11,13}$), 147.1 ($C^{17,22}$), 143.4 ($C^{27,31,39,43}$), 135.2 ($C^{15,20}$), 127.3 ($C^{14,19}$), 125.9 ($C^{16,21}$), 114.6 ($C^{26,38}$), 113.5 ($C^{28,30,40,42}$), 95.2 ($C^{25,37}$), 92.8 ($C^{24,36}$), 65.2 ($C^{35,47}$), 62.6 ($C^{55,57}$), 61.7 ($C^{63,68}$), 60.4 ($C^{10,12}$), 53.8 ($C^{5,6,7,9}$), 45.3 ($C^{4,8}$), 21.4 ($C^{32,33,44,45}$), 14.2 ($C^{56,58}$). **MALDI-TOF** MS(+) m/z calc. for $C_{52}H_{61}N_5O_{10}$ 915.4418, found 916.4423 $[M+H]^+$.

L^3

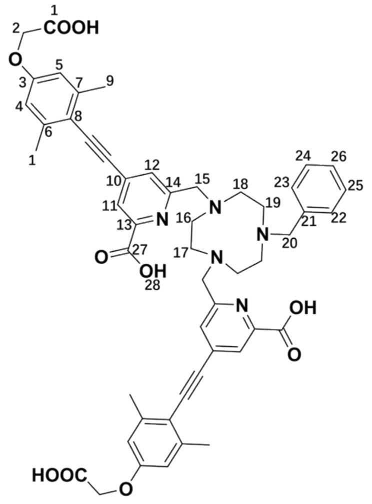

Compound **6** (28 mg, 0.03 mmol) was dissolved in dry MeCN (30 ml) and (bromomethyl)benzene (5.5 μL, 0.045 mmol) was added. K_2CO_3 (12 g, 0.09 mmol) was added and the mixture was stirred at 60 ˚C under nitrogen for 24 h. The reaction was allowed to cool to room temperature and filtered. The solvent was removed under reduced pressure. The crude tri-ester product was used in the next step without purification. An aqueous solution of sodium hydroxide 0.4 M (0.5 mL) was added to a solution crude product in methanol (0.5 mL). The mixture was stirred at 65 °C for 24 h. The reaction was monitored by LC-MS. After competition, the solvent was removed under reduced pressure and the resulting solid was purified by HPLC (*Method D*: ***t***t_R: 21.8 min). LCMS (ESI^+) m/z 893.822 $[M+H]^+$.($C_{51}H_{51}N_5O_{10}$ requires 893.99); **^{1}H NMR** (600 MHz, Methanol-d_4) δ 7.98 (d, J 1.4 Hz, 2H), 7.58 (d, J 1.4 Hz, 2H), 7.49 – 7.35 (m, 5H), 6.69 (s, 4H), 4.72 – 4.69 (m, 1H), 4.65 (s, 3H), 4.42 (s, 1H), 4.13 (s, 2H), 3.45 (s, 1H), 3.20 (s, 2H), 3.08 (s, 4H), 2.44 (s, 12H), 2.22 – 2.17 (m, 1H), 2.03 (q, J 7.0 Hz, 1H), 1.34 – 1.28 (m, 5H), 0.90 (t, J 7.0 Hz, 1H). **MALDI-TOF** MS(+) m/z calc. for $C_{51}H_{52}N_5O_{10}$ 894.3636, found 894.3627 $[M]^+$.

$[EuL^3]^-$ NH_4^+

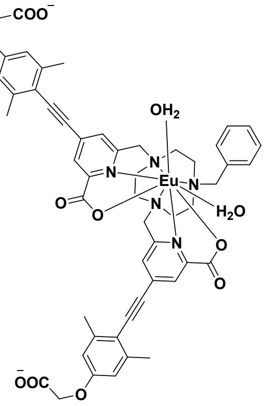

Ligand **L³** (6 mg, 6.7 μmol) was dissolved in methanol (0.5 mL) and europium trichloride hexahydrate (4 mg, 10μmol) added and the pH was readjusted to 6.5 by dropwise addition of aqueous sodium hydroxide solution (0.1 M) and dilute hydrochloric acid (0.1 M). The reaction mixture was stirred at 65 °C for 24 h. Aqueous ammonia solution (0.1 mL) was added to the reaction mixture and the white precipitate was filtered off. The solvent was removed under reduced pressure to give **[EuL³]** as a white solid that was used without further purification from the salt contaminant.

3-(Bromomethyl)-N-methylbenzamide,[2] was prepared according to the literature method.

L⁴

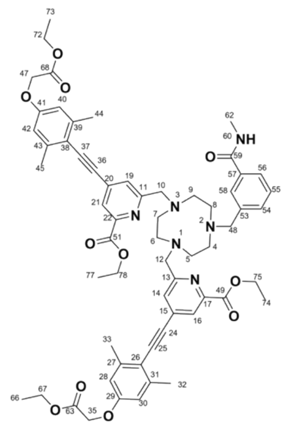

Compound **6** (25 mg, 0.027 mmol) and DIPEA (24 μL, 0.136 mmol) were dissolved in anhydrous CH_3CN (2.5 mL). 3-(Bromomethyl)-N-methylbenzamide, (7 mg, 0.030 mmol) was added and the mixture was stirred under nitrogen at 65 °C and the reaction monitored by LC-MS. The solvent was removed under reduced pressure and the crude material was purified by HPLC (*Method E*: t_R: 18.0 min) to give the tri-ester of L⁴ as a glassy solid (20 mg, 70%). **¹H NMR** (400 MHz, 296 K, Chloroform-*d*) *δ* 8.04 (s, 1H, NH), 7.82 (s, 2H, $H^{16,21}$), 7.78 – 7.31 (m, 4H, $H^{54,55,56,58}$), 7.43 (s, 2H, H^{14}), 6.64 (s, 4H, $H^{28,30,40,42}$), 4.63 (s, 4H, $H^{35,47}$), 4.15 (s, br. 4H, $H^{10,12}$), 4.28 (q, *J* 7.0 Hz, 8H, $H^{67,72,75,78}$), 4.15 (s, br. 2H, H^{48}), 4.02 – 3.35 (m, br. *ring* H), 2.97 (s, 2H, H^{62}), 2.46 (s, 12H, $H^{32,33,44,45}$), 1.31 (t, *J* 7.0 Hz, 6H, $H^{66,71}$), 1.21 (t, *J* 7.0 Hz, 6H, $H^{74,77}$). **¹³C NMR** (151 MHz, 296 K, $CDCl_3$) δ 168.7 ($C^{59,63,68}$), 164.7 ($C^{49,51}$), 158.4 ($C^{17,22,29,41}$), 148.2 ($C^{26,38}$), 143.2 ($C^{27,31,39,43}$), 135.9 (C^{57}), 134.3 (C^{53}), 132.8 (C^{54}), 128.8 (C^{56}), 127.6

($C^{14,16,19,21,58}$), 125.7 (C^{55}), 114.9 ($C^{15,20}$), 113.5 ($C^{28,30,40,42}$), 94.2 ($C^{25,37}$), 93.2 ($C^{24,36}$), 65.2 ($C^{35,47}$), 62.3 ($C^{75,78}$), 61.6 ($C^{67,72}$), 54.0 ($C^{10,12}$), 47.3 (ring C), 42.4 (ring C), 39.8 (C^{48}), 27.0 (C^{62}), 21.5 ($C^{31,33,44,45}$), 14.4 ($C^{74,77}$), 14.3 ($C^{66,71}$). **MALDI-TOF MS(+)** m/z 1064.254 $[M+H]^+$ ($C_{61}H_{70}N_6O_{11}$ requires 1063.262).

$[EuL^4]^-$ Na^+

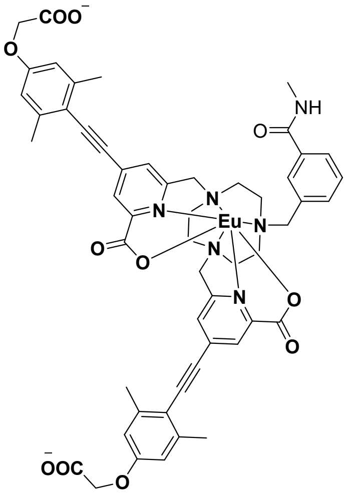

An aqueous solution of sodium hydroxide (0.4 M, 0.5 mL) was added to a solution of the diester of L^5 (20 mg, 18.8 µmol) in methanol (0.5 mL). The mixture was stirred at 60 °C for overnight. The reaction progress was monitored by LC-MS. **MALDI-TOF MS(+)** m/z calc. for $C_{53}H_{55}N_6O_{11}$. 951.3850, found $[M]^+$ 951.3867). Upon completion, aqueous hydrochloric acid (0.1 M) was added slowly until the pH reached 6.5. Europium chloride hexahydrate (2 mg, 5.5 µmol) was added and the pH readjusted to 6.5 by addition of aqueous sodium hydroxide solution (0.1 M). The reaction mixture was stirred at 60 °C for 18h, when solvent was removed under reduced pressure to afford a white solid, which was used directly without further purification from the NaCl salt impurity. **MALDI-TOF MS(+)** m/z 1101.293 ($C_{53}H_{52}EuN_6O_{11}$ requires 1101.290).

3-((N-Methylformamido)methyl)benzyl methanesulfonate,[3] was made by the literature route.

L^5 and its triethyl ester

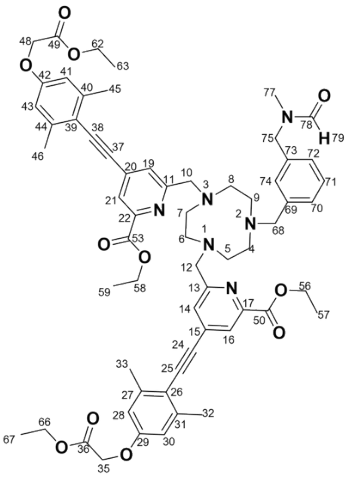

Compound **6** (32 mg, 0.035 mmol) and DIPEA (30 µL, 0.175 mmol) were dissolved in anhydrous CH_3CN (2.5 mL). ((N-Methylformamido)methyl)benzyl methanesulfonate (9 mg, 0.035 mmol) was added and the mixture was stirred under nitrogen at 65 °C and monitored by LC-MS. The solvent was removed under reduced pressure and the crude material was purified by HPLC (*Method E* : $\boldsymbol{t_R}$*:* 19.2 min) to give the triethyl ester of L^5 as a glassy solid (30 mg, 79%). **^{1}H NMR** (296 K, 400 MHz, Chloroform-*d*) δ 8.29 (s, 1H, H^{79}), 8.17 (s, 1H, H'^{79}), 7.87 (s, 2H, $H^{16,21}$), 7.43 (s, 2H, $H^{14,19}$), 7.40 (m, 2H, $H^{70,74}$), 7.10 (d, *J* 8.5 Hz, 1H, H^{72}), 6.95 (d, *J* 8.5, 1H, H^{71}), 6.64 (s, 4H, $H^{28,30,41,43}$), 4.63 (s, 4H, $H^{35,48}$), 5.52 (s, 2H, H^{75}), 4.50 – 4.33 (m, br. 6H, $H^{10,12,68}$), 4.29 (q, *J* 7.0, 4H, $H^{62,66}$), 4.22 (d, *J* 7.0 Hz, 4H, $H^{56,58}$), 3.78-3.35 (m, br. 12H, TACN-H), 2.92 (s, 3H, H^{77}), 2.77 (s, 3H, H'^{77}), 2.47 (s, 12H, $H^{32,33,45,46}$), 1.31 (t, *J* 7.0, 6H, $H^{63,67}$), 1.26 (t, *J* 7.0, 6H, $H^{57,59}$). **MALDI-TOF MS(+)** m/z 1078.276 $[M]^+$ ($C_{62}H_{73}N_6O_{11}$ requires 1078.289)

[EuL^5]

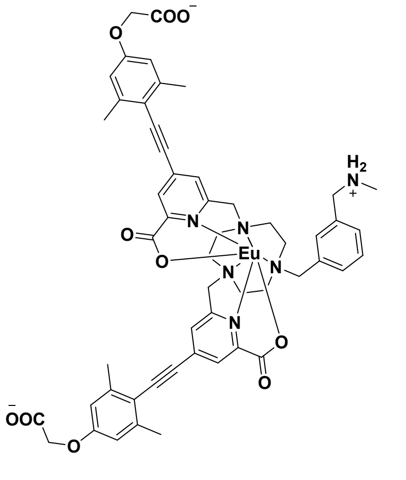

An aqueous solution of sodium hydroxide (0.4 M, 0.5 mL) was added to a solution of the triethyl ester of L^5 (3.2 mg, 2.97 µmol) in methanol (0.5 mL). The mixture was stirred at 60 °C overnight. The reaction was monitored by LC-MS. **MALDI-TOF MS(+)** m/z calc. for $C_{53}H_{57}N_6O_{10}$. 937.4057, found $[M]^+$ 937.4030. Upon completion, aqueous hydrochloric acid (0.1 M) was added slowly until the pH reached 6.5. Europium chloride hexahydrate (1.5 mg, 4.09 µmol) was added and the pH readjusted to 6.5 by addition of aqueous sodium

hydroxide solution (0.1 M). The reaction was stirred at 60 °C for 18h. Aqueous ammonia solution (0.1 mL) was added and the white precipitate was removed by filtration. The solvent was removed under reduced pressure to give the complex as a colourless solid (0.8 mg, 24 %). **MALDI-TOF (ESI$^+$)** m/z 1083.3214 ($C_{53}H_{52}EuN_6O_{10}{}^{153}Eu$ requires 1085.2957); λ_{exc} (H_2O) = 347 nm; τ (H_2O) = 0.64 ms; τ (D_2O) = 0.75 ms, *q* = 0.

SI References

[1] H. Li, S. L.-W. Ng, D. J. Black, W. Han, R. Pal, D. Parker, *Chem. Sci*. 2025, **16**, 14544-14552.

[2] D. G. Smith, G.-l. Law, B. S. Murray, R. Pal, D. Parker, K.-L. Wong, *Chem. Commun*. 2011, **47**, 7347-7349.

[3] M. Starck, R. Pal, D. Parker, *Chem.– Eur. J*. 2016, **22**, 570-580.

[4] S. Shah, M. Kulkarni, R. Mashelkar, *J. Controlled Release* 1991, **15**, 121-131.

[5] E. R. Neil, M. A. Fox, R. Pal, D. Parker, *Dalton Trans*. 2016, **45,** 8355-8366.

SI Figures

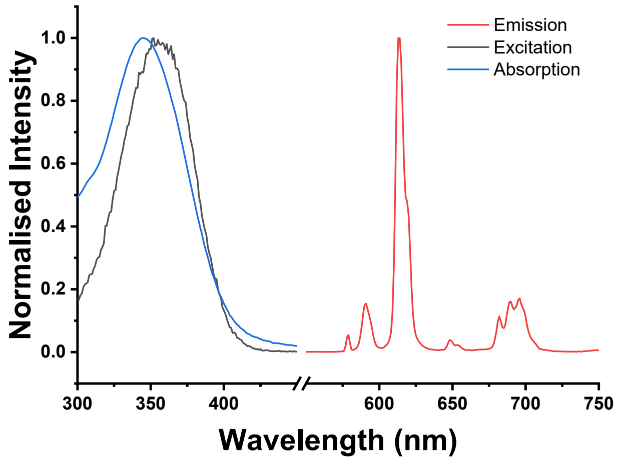

Fig. S1 Absorption, excitation and emission spectra for $[EuL^3]^-$ (1:99/DMSO: H_2O-pH 7.4 HEPES buffer, 295 K, λ_{exc} 350 nm).

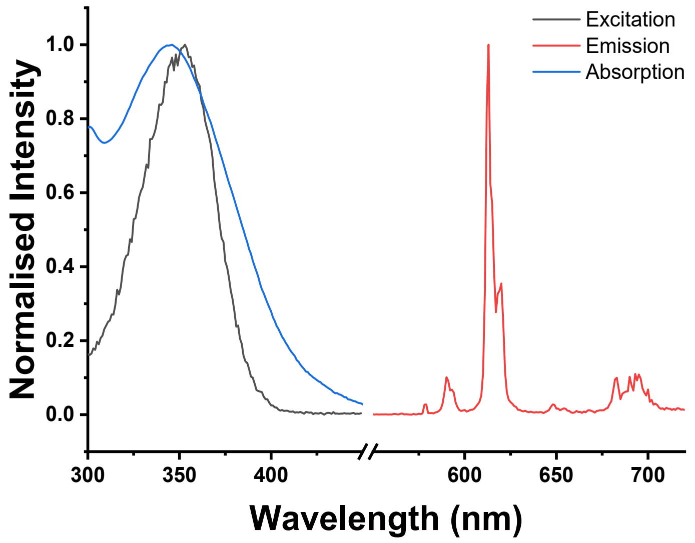

Figure S2 Absorption, excitation and emission spectra for [EuL^5] (1:99/DMSO: H_2O-pH 7.4 HEPES buffer, 295 K, λ_{exc} 350 nm).

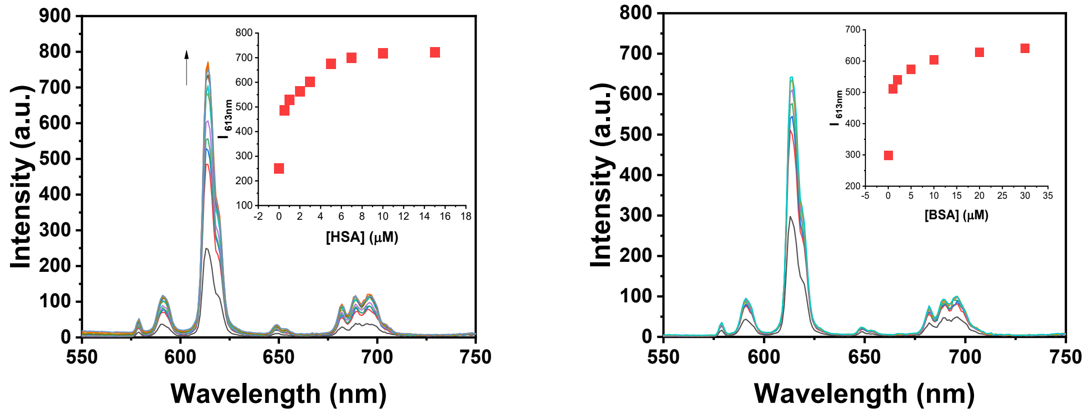

Figure S3 Change of the total emission intensity upon addition of HSA (*left*) and BSA (*right*) (to [EuL^4]$^-$;([EuL^4]$^-$ 5 μM, the inset assumes a 1: 1 binding isotherm, (1:99/DMSO: H_2O-pH 7.4 HEPES buffer, 295 K, λ_{exc} 350 nm).

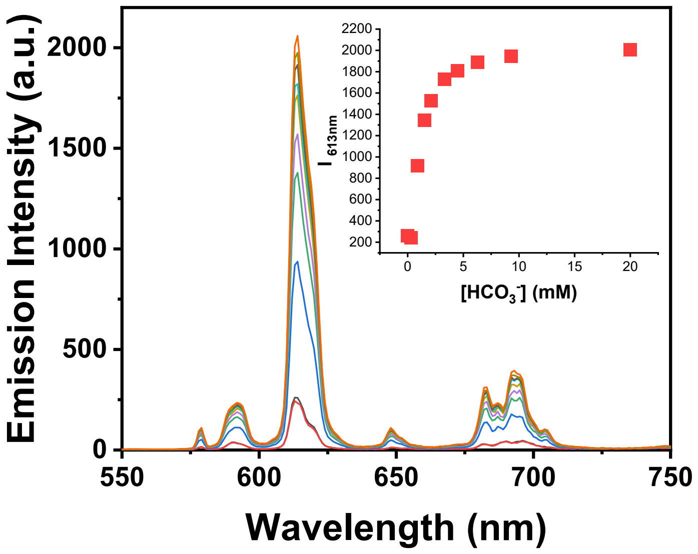

Figure S4 Change of the total emission intensity upon addition of sodium bicarbonate to $[EuL^4]^-$;($[EuL^4]^-$ 5 μM, the inset assumes 1: 1 binding stoichiometry, 1:99/DMSO: H_2O-pH 7.4 HEPES buffer, 295 K, λ_{exc} 350 nm).

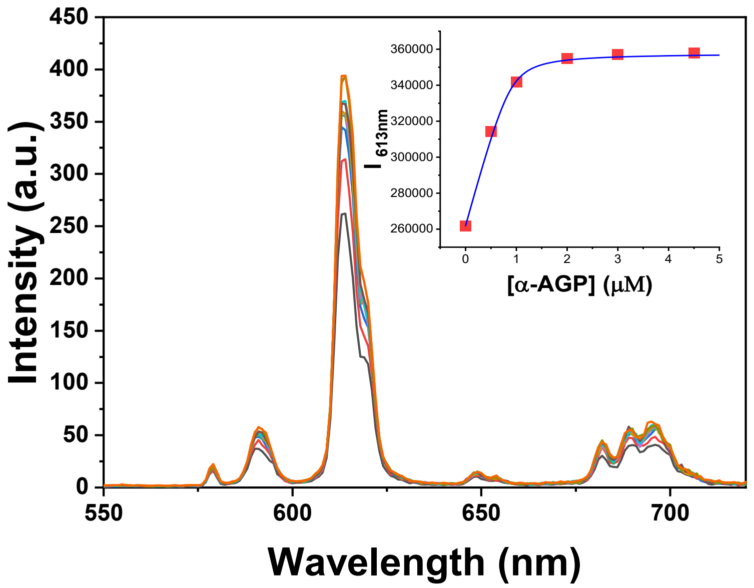

Figure S5 Change of the total emission intensity upon addition of α_1-AGP to $[EuL^4]^-$;($[EuL^4]^-$ 5 μM, the inset assumes a 1: 1 binding isotherm, (1:99/DMSO: H_2O-pH 7.4 HEPES buffer, 295 K, λ_{exc} 350 nm).

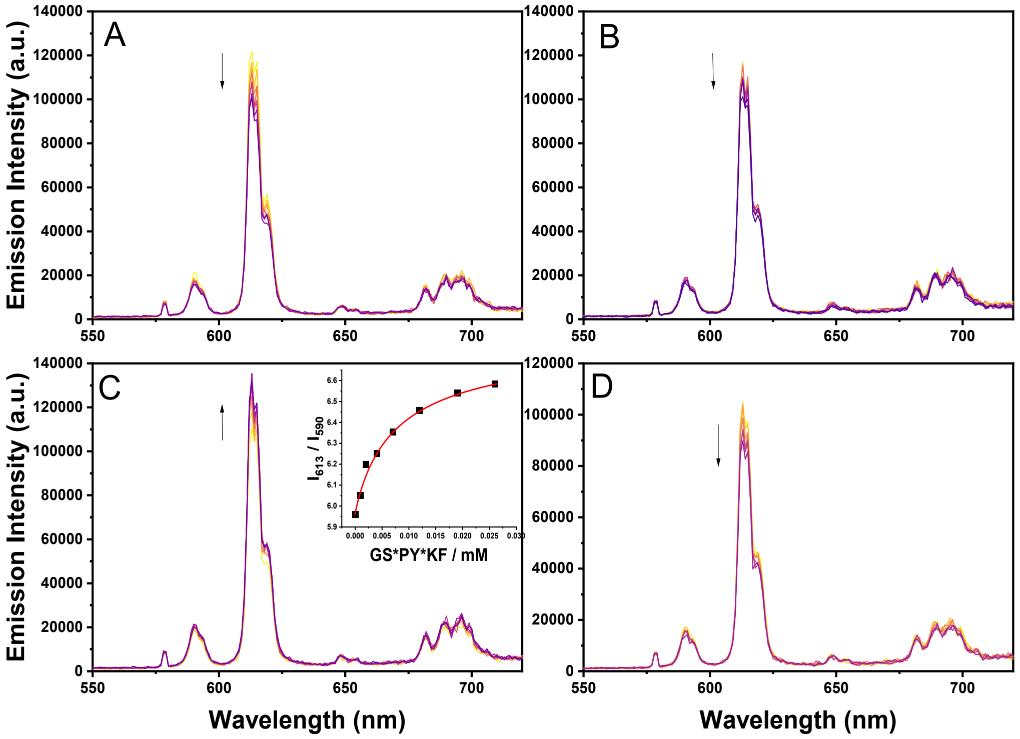

Figure S6 Change of the total emission intensity upon addition of phosphorylated peptides to $[EuL^4]^-$;(A) GS*PFKF, (B) GAPY*KF, (C) GS*PY*KF, (D) GAPYKF. ($[EuL^4]^-$ 5 μM, the inset assumes a 1:1 binding isotherm, (66%MeOH: 33 % H_2O/10 mM HEPES buffer pH 7.4/1% DMSO, 295 K, λ_{exc} 350 nm).

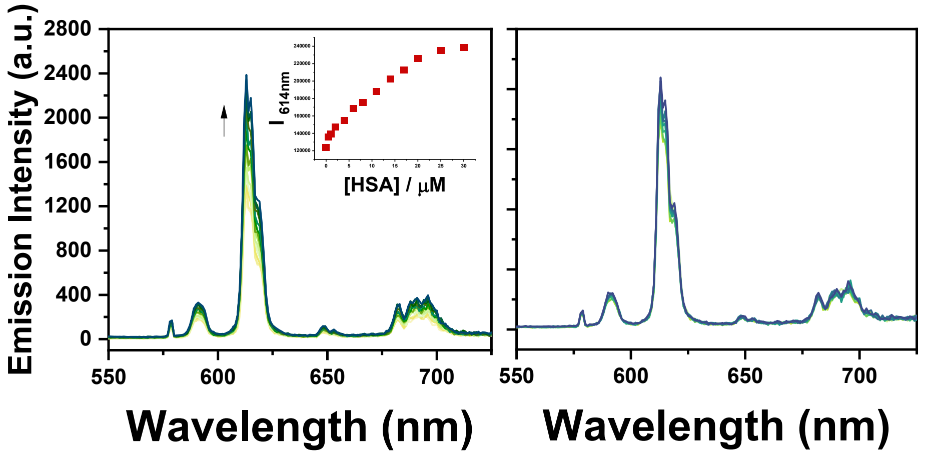

Figure S7 Variation of the Eu(III) emission spectrum for $[EuL^5]$, following addition of HSA (*left*) and BSA (*right*) (1% DMSO in aqueous HEPES buffer, pH 7.40, 0-30 μM protein, 5 μm complex, 295 K, λ_{exc} 340 nm). There is no significant change in the integrated emission intensity ratio for the $\Delta J = 1$ and $\Delta J = 2$ transitions. The variation of emission intensity data at 615 nm with added HSA concentration could not be fitted satisfactorily to a simple 1:1 binding isotherm.

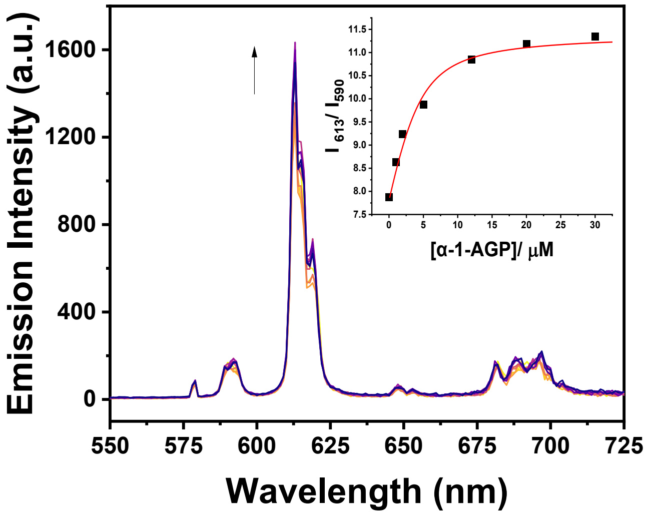

Figure S8 Variation of the Eu(III) emission spectrum for [EuL^5]$^-$, following addition of alpha-1-acid glycoprotein (1% DMSO in aqueous HEPES buffer, pH 7.40, 0-30 μM α-1-AGP, 5 μm complex, 295 K, λ_{exc} 350 nm). The inset shows the ratiometric variation of band intensities for 590 nm vs 613 nm, and a binding constant (log *K* = 5.84 (05)) was estimated, assuming 1:1 stoichiometry.

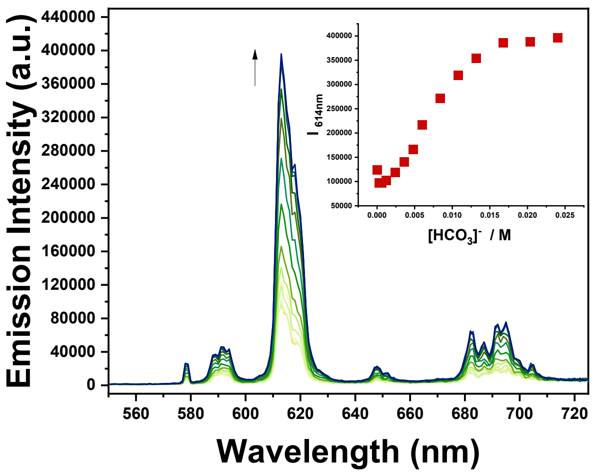

Figure S9 Variation of the Eu(III) emission spectrum for [EuL^5], following addition of sodium bicarbonate (1% DMSO in aqueous HEPES buffer, pH 7.40, 0-25 mM sodium bicarbonate, 5 μm complex, 295 K, λ_{exc} 340 nm). The inset shows the variation at 614 nm.

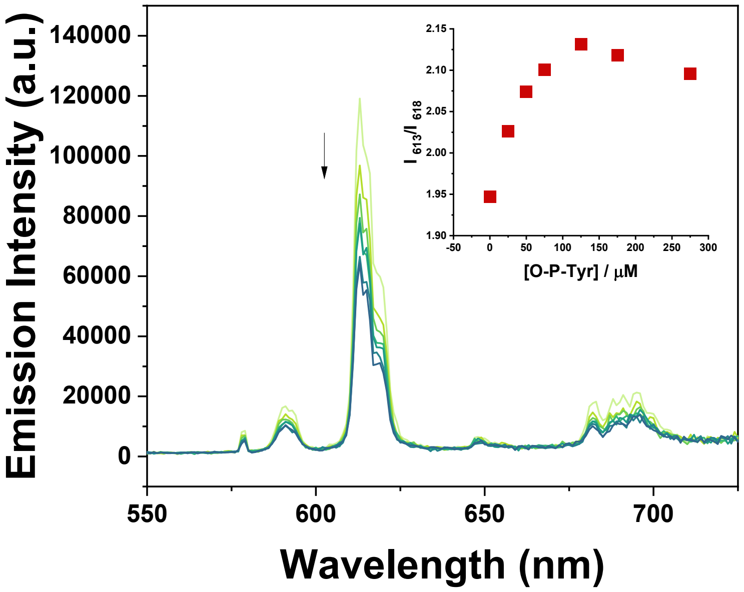

Figure S10 Variation of the Eu(III) emission spectrum for [EuL^5], following addition of O-P-Tyr (1% DMSO in aqueous HEPES buffer, pH 7.40, 0-300 µm O-P-Tyr, 5 µm complex, 295 K, λ_{exc} 340 nm). The inset shows the variation of the intensity ratio at 613 nm and 618 nm.

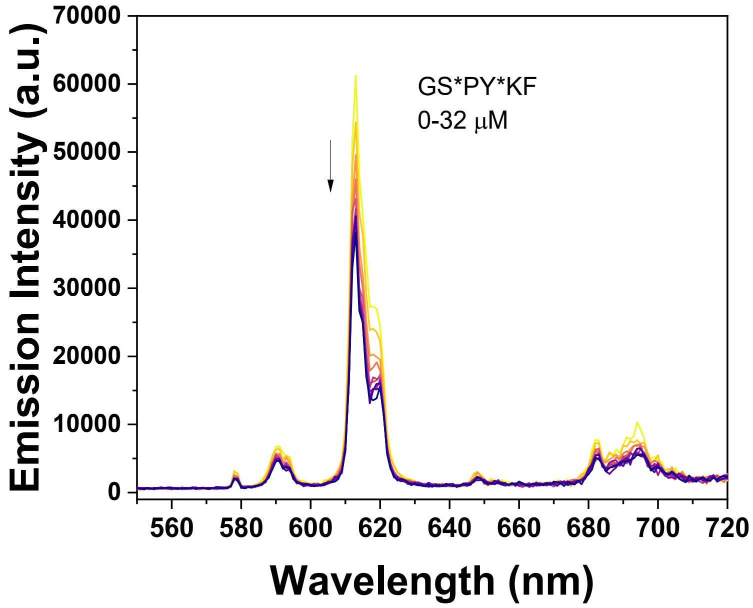

Figure S11 Variation in the Eu(III) emission spectral profile for [EuL^5] as a function of added GS*PY*KF. (1% DMSO ; 10 mM HEPES buffer pH 7.4 , 5 µM complex, λ_{exc} 340 nm, 295 K).

Figure S12 ^{1}H NMR spectrum ($CDCl_3$, 400 MHz, 295 K) of compound **5**

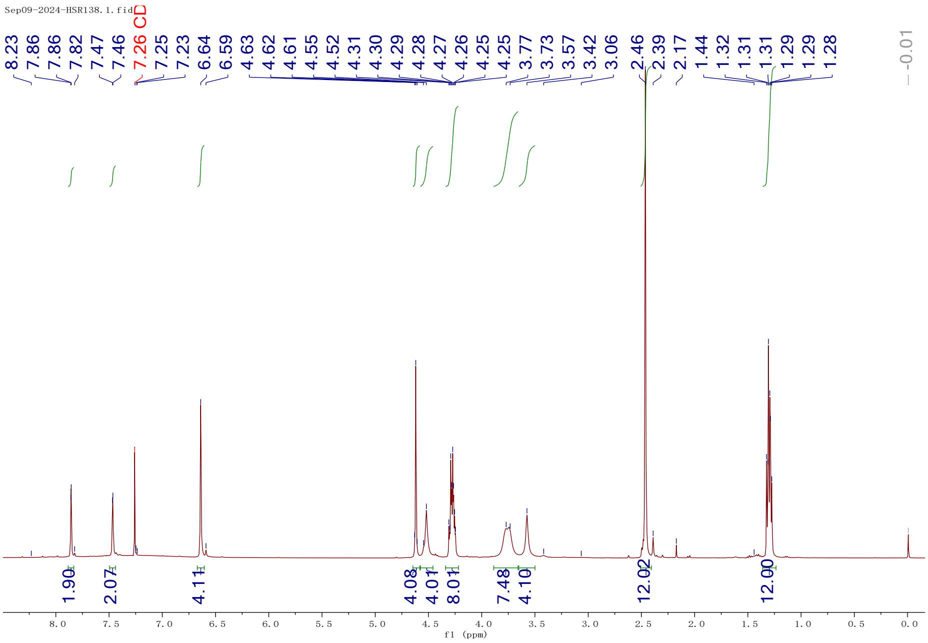

Figure S13 ^{13}C NMR spectrum ($CDCl_3$, 400 MHz, 295 K) of compound **5**

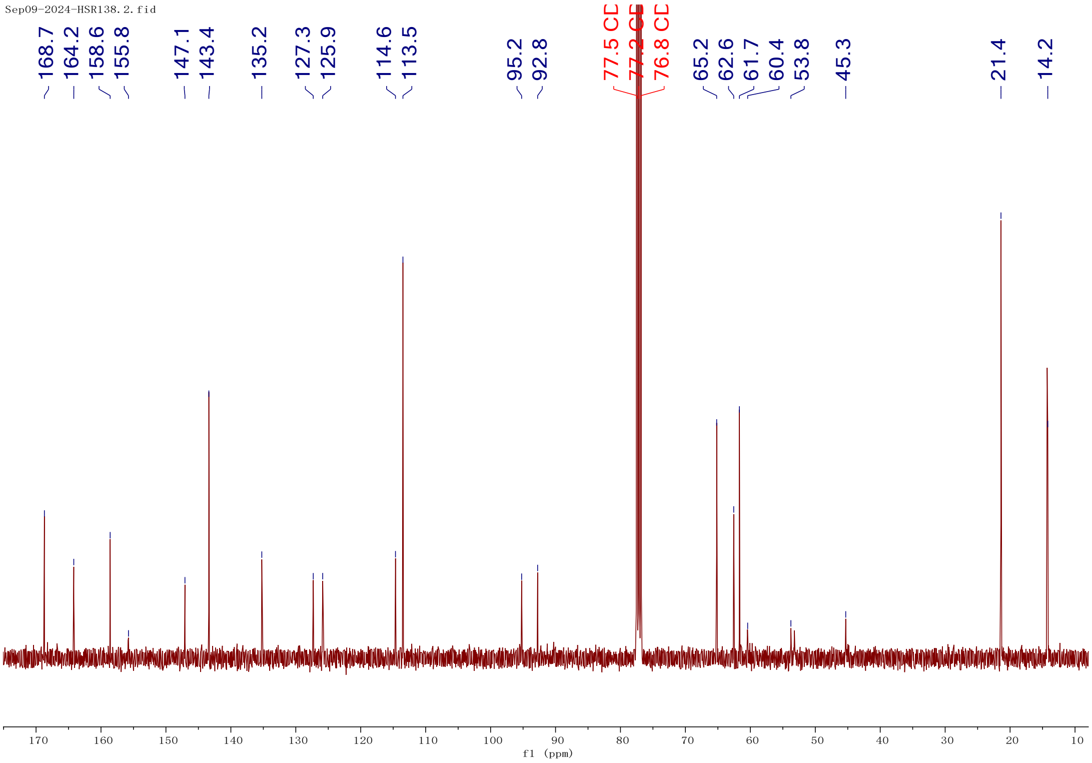

Figure S14 ^{1}H NMR spectrum ($CDCl_3$, 400 MHz, 295 K) of **L^3**

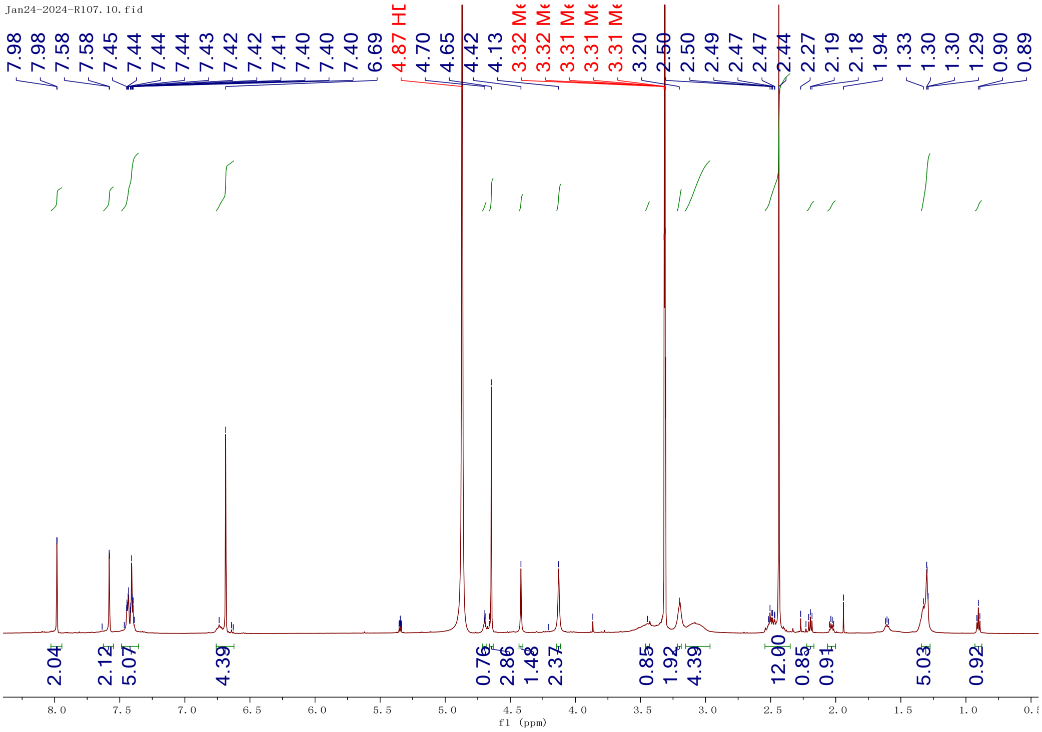

Figure S15 ^{1}H NMR spectrum ($CDCl_3$, 400 MHz, 295 K) of triethyl ester of L^4

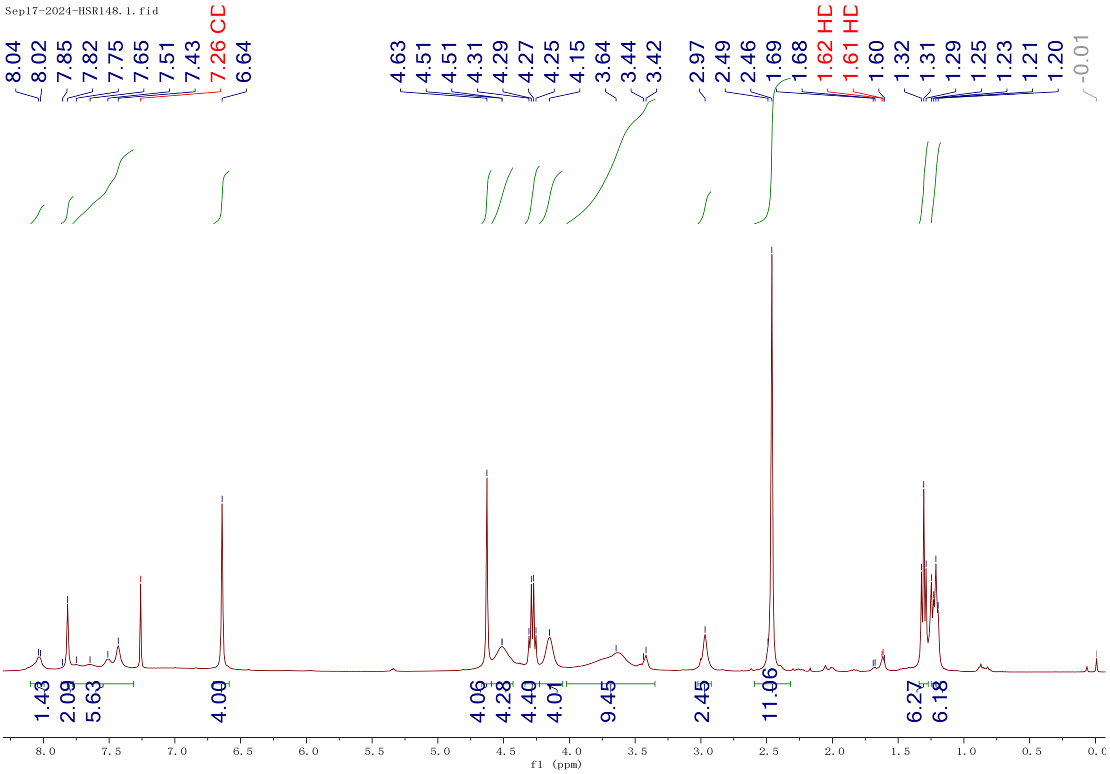

Figure S16 ^{13}C NMR spectrum ($CDCl_3$, 400 MHz, 295 K) of triethyl ester of L^4

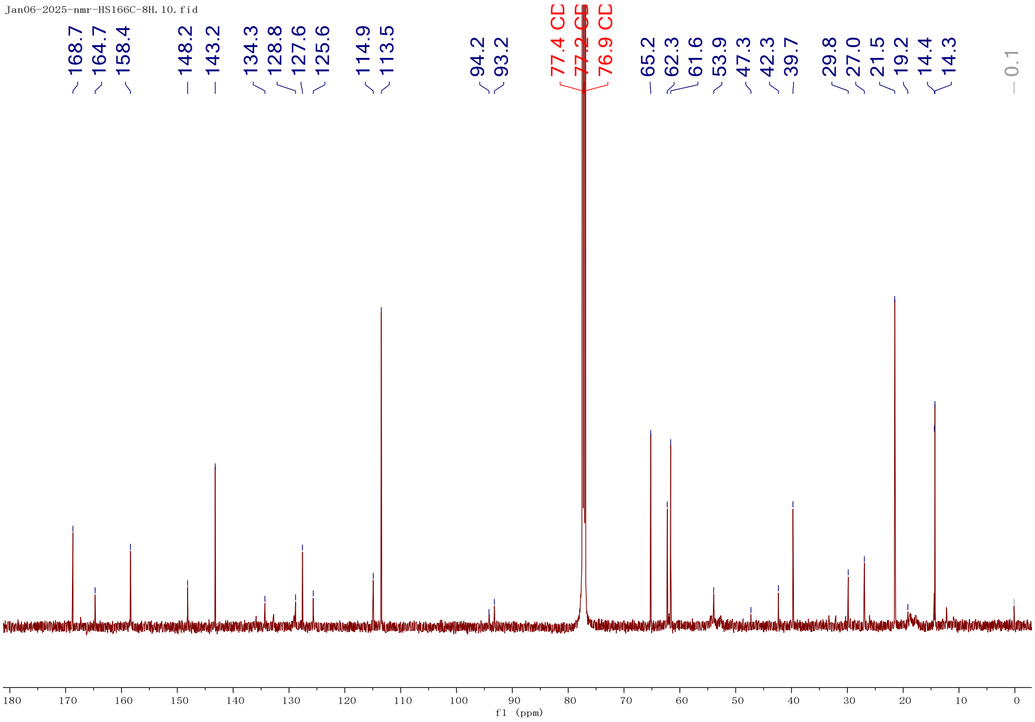

Figure S17 ^{1}H NMR spectrum ($CDCl_3$, 400 MHz, 295 K) of triethyl ester of L^5

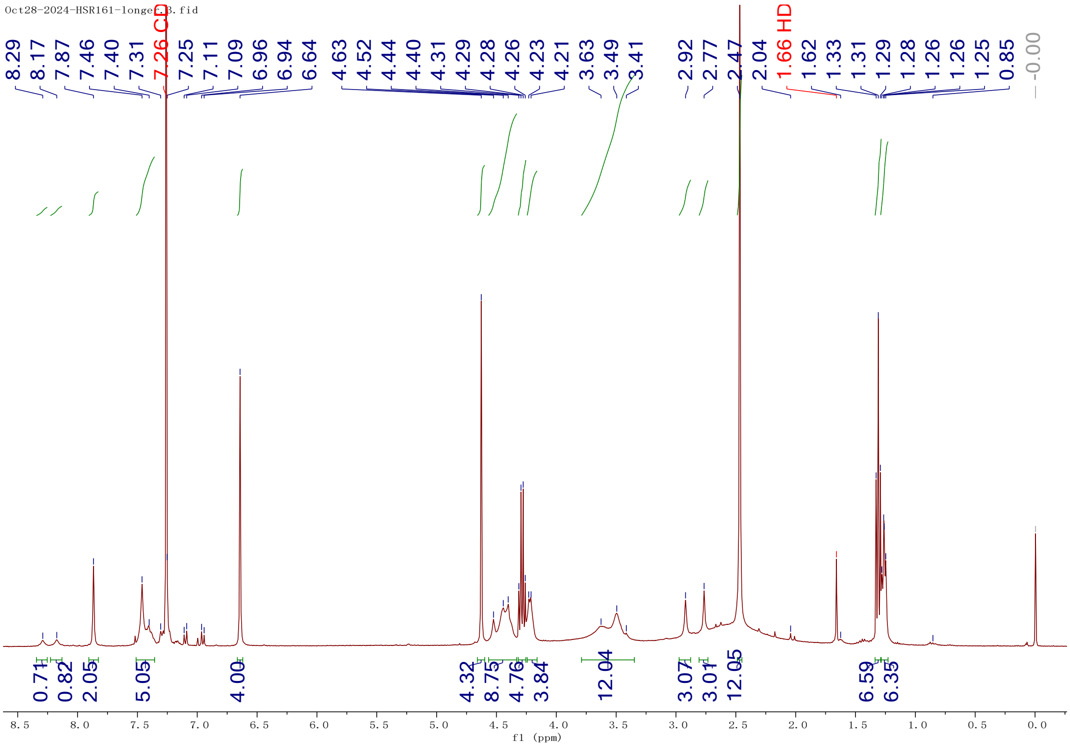

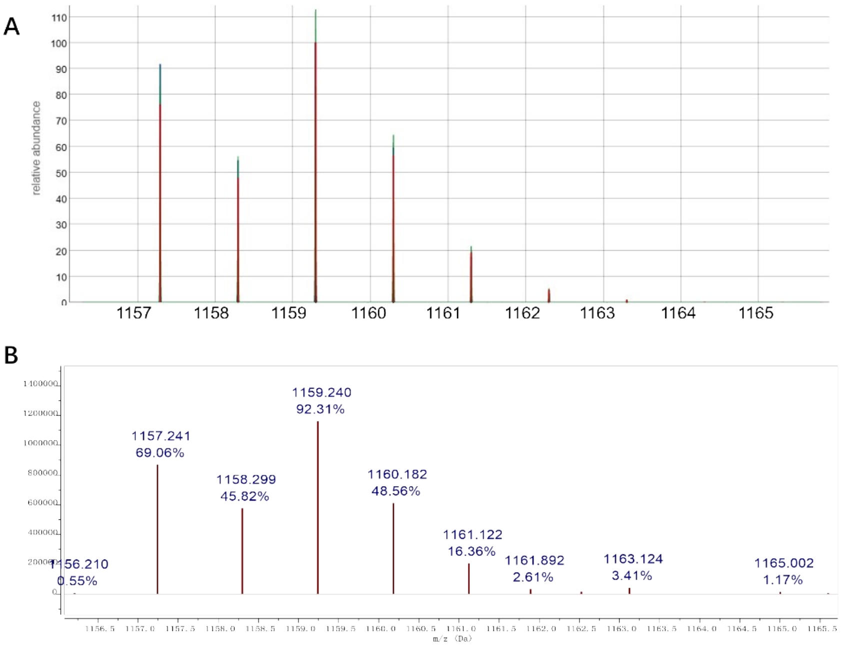

Fig. S18 LC−ESMS analysis of [EuL^4]/acetate adducts obtained in negative ion mode. Isotope pattern: (A) calculated; (B) observed in the [(M-H)+CH_3CO_2]$^-$ complex adduct.

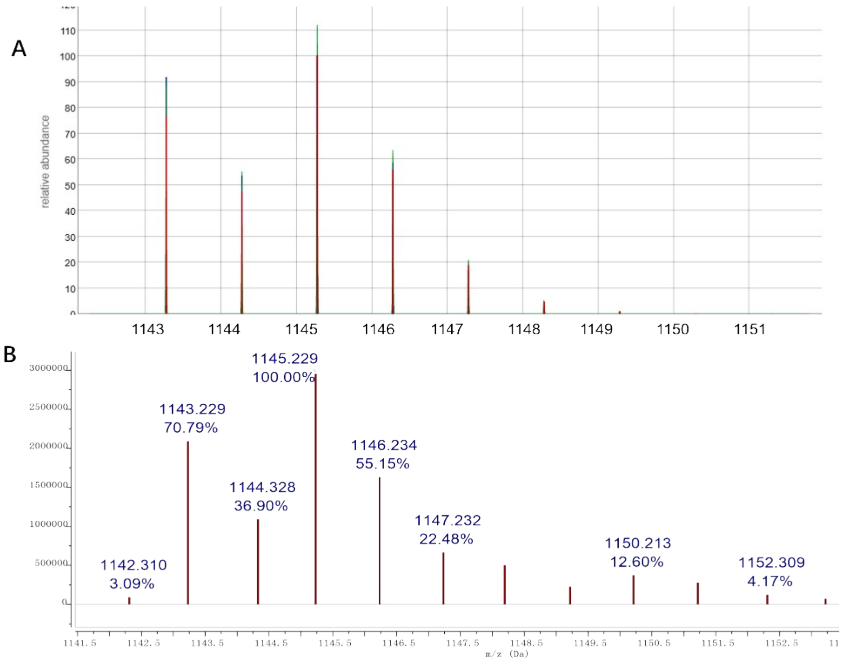

Fig. S19 LC−ESMS analysis of the [EuL^4] /formate adduct, obtained in negative ion mode. Isotope patterns: (A), calculated;(B), observed in the [(M-H)+$HCOO^-$]$^-$ adduct.

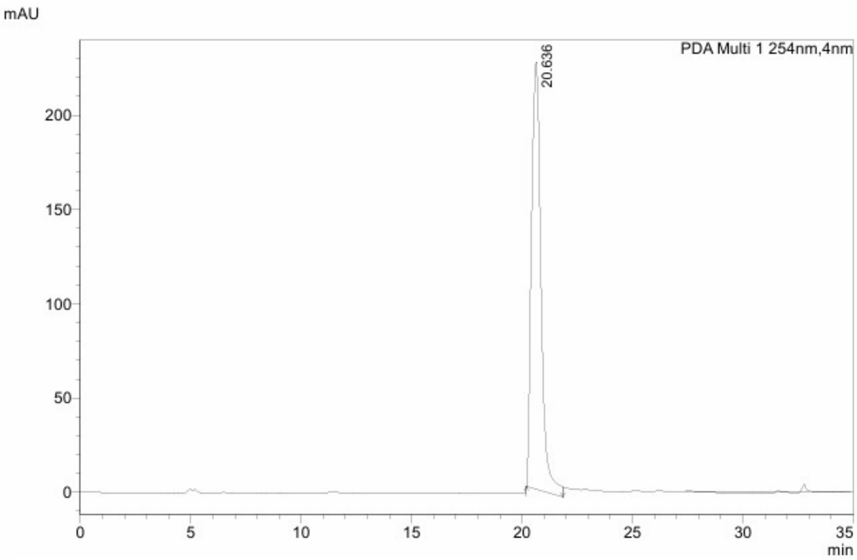

Fig. S20 HPLC trace (254 nm) for L[3] on an XBridge Prep C18 column (19 x 100 mm, 5 µm), 25 °C. Isocratic elution 58:42 H_2O/MeCN; flow rate 5.0 mL/min (t_r 20.6 min with a purity of 99.5% (by area percent integration at 254 nm)).

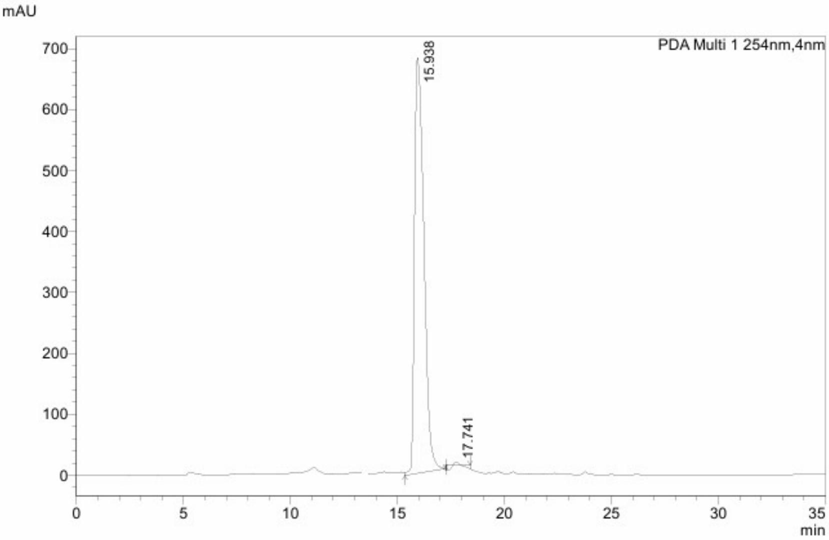

Fig. S21 HPLC trace (254 nm) for L[4] on an XBridge Prep C18 column (19 x 100 mm, 5 µm), 25 °C. Isocratic elution 64:36 H_2O/MeCN; flow rate 5.0 mL/min (t_r 16.0 min with a purity of 99% (by area percent integration of the signal observed at 254 nm)).

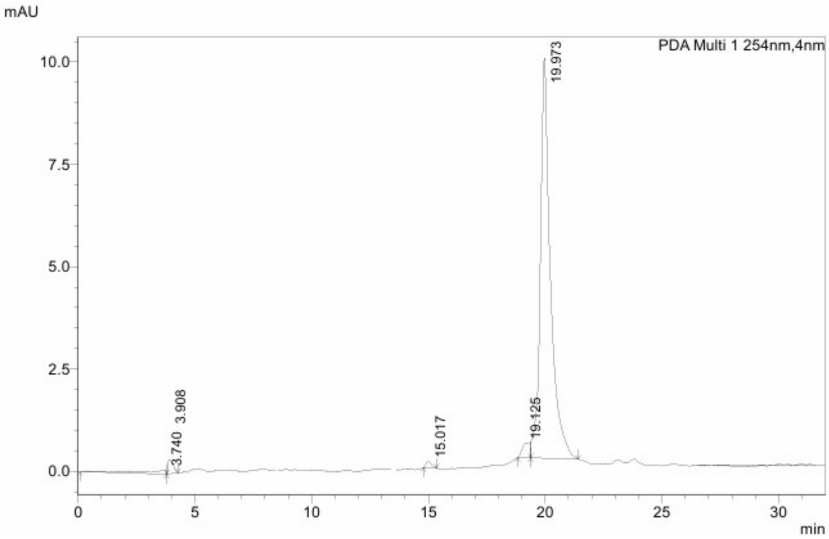

Fig. S22 HPLC trace (254 nm) for L^5 on an XBridge Prep C18 column (19 x 100 mm, 5 µm), 25 °C. Isocratic elution 60:40 H_2O/MeCN; flow rate 5.0 mL/min (t_r 20.0 min with a purity of 97% (by area percent integration at 254 nm).).
